# Nutrient intake disparities in the US: modeling the effect of food substitutions

**DOI:** 10.1186/s12937-018-0360-z

**Published:** 2018-05-17

**Authors:** Zach Conrad, LuAnn K. Johnson, James N. Roemmich, WenYen Juan, Lisa Jahns

**Affiliations:** 1 Grand Forks Human Nutrition Research Center, US Department of Agriculture, Agricultural Research Service, 2420 2nd Ave. N, Grand Forks, ND 58203 USA; 2 Center for Food Safety and Applied Nutrition, US Department of Health and Human Services, Food and Drug Administration, 5001 Campus Drive, College Park, MD 20740 USA

**Keywords:** Diet quality, Nutrient intake, Food security, Food assistance, Diet model

## Abstract

**Background:**

Diet quality among federal food assistance program participants remains low, and little research has assessed the diet quality of food insecure non-participants. Further research is needed to assess the extent to which food substitutions can improve the nutritional status of these vulnerable populations. Substituting egg dishes for other commonly consumed dishes at certain eating occasions may be an effective strategy for improving the daily nutrient intake among these groups. Eggs are rich in many important nutrients, and are low-cost and part of a wide range of cultural food menus, which are important considerations for low-income and ethnically diverse populations. To help guide the focus of targeted nutrition interventions and education campaigns for vulnerable populations, the present work begins by 1) estimating the prevalence of nutrient inadequacy among these groups, and then models the effect of consuming egg dishes instead of commonly consumed dishes at each eating occasion on 2) the prevalence of nutrient inadequacy, and 3) the mean intake of nutrients.

**Methods:**

Dietary data from 34,741 adults ≥ 20 y were acquired from the National Health and Nutrition Examination Survey, 2001–2014. Diet pattern modeling was used to substitute commonly consumed egg dishes for commonly consumed main dishes at breakfast, lunch, and dinner. National Cancer Institute usual intake methods were used to estimate the prevalence of inadequate intake of 31 nutrients pre- and post-substitution, and a novel index was used to estimate change in intake of all nutrients collectively.

**Results:**

Substituting eggs for commonly consumed main dishes at lunch or dinner did not change total daily nutrient intake for each group (*P* > 0.05), but decreased the prevalence of vitamin D inadequacy by 1–4 percentage points (*P* < 0.01). Substituting eggs for commonly consumed foods at breakfast increased the prevalence of folate inadequacy by 8–12 percentage points among each group (*P* < 0.01).

**Conclusions:**

When making food substitutions to increase nutrient intake, eating occasion should be an important consideration. Further research is needed to better understand how food substitutions affect diet costs, which may be an important driver of food purchasing decisions among low income individuals with limited food budgets.

**Electronic supplementary material:**

The online version of this article (10.1186/s12937-018-0360-z) contains supplementary material, which is available to authorized users.

## Background

Federal food assistance programs in the US have expanded in scale and number over the past century, and are now a cornerstone of the national nutrition agenda. These programs represent essential tools to reduce food insecurity and improve the diet quality of low-income individuals and households across the country.

The largest of these programs, in federal financial outlays and the number of individuals participating, are the Supplemental Nutrition Assistance Program (SNAP) and Special Supplemental Nutrition Program for Women, Infants, and Children (WIC). SNAP, formerly known as the “Food Stamp Program”, provides financial benefits to eligible households to purchase foods for at-home consumption. Eligibility is determined primarily by household income at or below 130% of the poverty level [1]. Over 45 million individuals participated in SNAP in 2016, [2] with annual program costs exceeding $80 billion [3]. In contrast to SNAP, WIC provides financial benefits to eligible individuals to purchase a select suite of approved foods [4]. Eligibility is determined by a categorical requirement (pregnant, post-partum, or breastfeeding women; infants; and children), income requirement (state-specific income-to-poverty ratio threshold), and nutrition risk requirement (individuals deemed to be at risk of nutrition-related poor health outcomes). Approximately 9.5 million individuals participated in WIC in 2016, [4] with a program cost of $6.6 billion [3].

Low diet quality is the leading cause of mortality in the US [5]. Income is an important moderator of diet quality, with lower income individuals exhibiting lower quality diets than their higher income counterparts [6, 7]. SNAP and WIC participants, in particular, generally exhibit low quality diets [8, 9] and high rates of cardiometabolic mortality [10]. Yet little research has assessed the diet quality of food insecure individuals who are not participating in either SNAP or WIC. Further efforts are needed to address this important research gap and to assess interventions to improve the nutritional status of these vulnerable populations.

Evidence suggests that moderate diet and lifestyle changes can be more easily adopted and sustained than more drastic changes, and can ultimately elicit meaningful health improvements [11–13]. Following this principle, one approach to improving diet quality is to counsel individuals to make healthier food choices one meal at a time. Several studies have found that the greatest amount of calories is consumed at dinner, yet the intake of micronutrients at each eating occasion was not examined [14–16]. Others have examined the micronutrient contribution of individual foods to daily nutrient intakes, such as beef, [17] dairy, [18–20] breakfast cereal, [21] orange juice, [22] potatoes, [23] avocados, [24] and canned fruits and vegetables, [25] yet evidence is lacking on how specific food choices at each eating occasion (i.e. breakfast, lunch, and dinner) contribute to daily nutrient intake.

Substituting egg dishes for other commonly consumed dishes at certain eating occasions may be an effective strategy for improving the daily nutrient intake among vulnerable populations. Eggs are rich in many important nutrients, [26–28] increase the bioavailability of co-consumed carotenoids [29] and vitamin E, [30] and promote positive health outcomes at all ages and all stages of life [30, 31]. In a recent study, pregnant egg consumers were much more likely to meet daily choline intake recommendations than pregnant non-egg consumers [32]. And eggs are low-cost and part of a wide range of cultural food menus, [31] which are important considerations for low-income and ethnically diverse populations.

To help guide the focus of targeted nutrition interventions and education campaigns for individuals participating in federal food assistance programs and those with low food security, the present work begins by 1) estimating the prevalence of nutrient inadequacy among these groups, and then models the effect of consuming egg dishes instead of commonly consumed dishes at each eating occasion on 2) the prevalence of nutrient inadequacy, and 3) the mean intake of nutrients.

## Methods

### Dietary data

Data on individual-level food and nutrient intake, SNAP and WIC participation, food security status, and other characteristics were acquired from the National Health and Nutrition Examination Survey (NHANES), waves 2001–2002, 2003–2004, 2005–2006, 2007–2008, 2009–2010, 2011–2012, and 2013–2014 [33]. NHANES is a continuous, cross-sectional survey that collects data on demography and health behaviors from a sample of ~ 5000 individuals per year, and data are released in two-year cycles. Data on food and nutrient intake were acquired from What We Eat In America (WWEIA), the dietary component of NHANES. Individuals complete a 24-h recall (24HR) administered by a trained interviewer using United States Department of Agriculture’s (USDA) Automated Multiple Pass Method, [34] and a subset of the study population (~ 80%) completes a subsequent 24HR by telephone on a non-consecutive day. Food security status was assessed by trained NHANES staff using the US Food Security Module [35].

### Study populations

All individuals 20+ y with dietary recalls that were considered reliable by trained NHANES staff were included in this study (*n* = 34,741). Respondents were categorized as SNAP participants, WIC participants, food insecure non-participants, and food secure non-participants. SNAP participants were identified as individuals who reported that someone in their household was participating in SNAP at the time of the survey (*n* = 4020), and WIC participants were identified as individuals who reported that they were participating in WIC at the time of the survey (*n* = 636). Individuals who reported participating in SNAP and WIC at the time of the survey (*n* = 209) were categorized as WIC participants in order to maintain the relative homogeneity of this group compared to the SNAP group (due to the more restrictive eligibility requirements of WIC). Individuals who did not report participating in SNAP or WIC but reported low or very low food security were categorized as food insecure non-participants (*n* = 3631), and individuals who did not report participating in SNAP or WIC and reported marginal or full food security were categorized as food secure non-participants (*n* = 26,454).

### Identifying main dishes at each eating occasion

For each individual food reported consumed in WWEIA 2001–2014, the eating occasion and time of day each food was consumed was reported in either English or Spanish. Foods reported consumed at breakfast, lunch, or dinner were grouped into main dish categories primarily according to the categorization scheme used in the Food and Nutrient Database for Dietary Studies (FNDDS; Additional file 1: Table S1) [36]. A total of 17 main dish categories were identified: beef dishes, pork dishes, bacon, poultry dishes, other meat dishes, seafood dishes, sandwiches, sausages, soups, breads, sweet baked goods, pancakes and waffles, oatmeal, breakfast cereal, pasta, Mexican dishes, and pizzas and calzones. Additionally, six egg dishes were identified: whole eggs, scrambled eggs and omelets, egg sandwiches, egg soups, frozen egg dishes, and quiches. A composite nutrient profile was created for each main dish and each egg dish by averaging the nutrient content per gram weight of each of the foods in each dish category.

### Diet modeling

A diet model was constructed to estimate the effects on daily nutrient intake if egg dishes were substituted for commonly consumed main dishes at each eating occasion (i.e. breakfast, lunch, and dinner). Commonly consumed dishes at each eating occasion among each group (i.e. SNAP participants, WIC participants, food insecure non-participants, and food secure non-participants) were identified by frequency of reported consumption (i.e. percent of individuals reporting consumption of each dish). The diet model replaced the most commonly consumed main dish with the most commonly consumed egg dish at each eating occasion for each individual in each group, on a gram weight basis. For example, if the most commonly consumed main dish among SNAP participants at breakfast was bread, and the most commonly consumed egg dish among SNAP participants at breakfast was scrambled eggs, then for each SNAP participant the amount of bread consumed (in grams) was replaced with the same gram weight of scrambled eggs.

### Total nutrient index

A total nutrient index was developed to provide a summary estimate of how much nutrient intake changed (for all nutrients collectively) when egg dishes were consumed instead of the most commonly consumed dishes at each eating occasion. This index is interpreted as the mean percent change in nutrient intake from baseline intake to modeled intakes. The total nutrient index was calculated in two steps: first, by estimating the percent change in intake from baseline for each individual nutrient, and then 2) computing the mean percent change in nutrient intake across all nutrients. Nutrients which Americans are encouraged to limit, including sodium and saturated fat, were reverse scored prior to computing the total nutrient index so that greater values represented lower percent change from baseline. To ensure that more accurately measured nutrient intakes had greater influence on the index, the total nutrient index was weighted by the standard error of each nutrient, so that nutrients with greater standard errors received less weight in the index. The total nutrient index can be expressed as:

$$ Total\ nutrient\ index\kern0.5em =\kern0.5em \frac{\sum_{i=1}^N{C}_i/{se}_i}{\sum_{i=1}^N{se}_i} $$

where *C* is the percent change in the intake of a given nutrient (*i*) weighted by its standard error (*se*), and *N* is the number of nutrients included in the index.

Sensitivity analyses were used to test the sensitivity of the total nutrient index to potentially influential individual nutrients. In these analyses, nutrients with the greatest change from baseline were iteratively removed from the index and compared against the original index.

### Statistical analyses

Daily nutrient intake was estimated at baseline (i.e. reported daily intake) and for three modeled intakes: substituting egg dishes for the most commonly consumed main dishes at breakfast, lunch, and dinner. The percent of individuals in each group not meeting age-and sex-specific daily intake recommendations, as well as the mean daily intake of each nutrient, were estimated using the National Cancer Institute’s (NCI) usual intake methodology [37]. The NCI method uses information from two 24HRs to estimate within-person variation using the SAS macros MIXTRAN and DISTRIB. MIXTRAN uses logistic regression with a person-specific random effect to estimate the probability of consumption, accounting for the day of intake and whether it was a weekday or weekend; DISTRIB estimates daily intake; and the results from both macros are linked with a correlated person-specific random effects model.

The percent of individuals not meeting daily intake recommendations was estimated for the 16 nutrients with an Estimated Average Requirement (EAR; vitamins A, B_6_, B_12_, C, D, E; thiamin; riboflavin; niacin; folate; calcium; iron; magnesium; and zinc), an Upper Limit (UL; sodium), or a Dietary Guidelines for Americans 2015–2020 (DGA) recommendation (saturated fatty acids; Additional file 2: Table S2). Mean nutrient intakes were estimated for all nutrients, additionally including 12 nutrients without a recommended intake amount: fiber, eicosapentanoic acid, docosahexaenoic acid, α-linolenic acid, vitamin K, choline, potassium, α-carotene, β-carotene, β-cryptoxanthin, lutein and zeaxanthin, and lycopene. Mean intakes were also estimated for total protein, carbohydrate, and fat. The balanced repeated replication method was used to estimate standard errors while accounting for the complex sampling design of NHANES.

All analyses were adjusted for age (20–30, 31–50, 51–70, 71+), gender, race-ethnicity (non-Hispanic white, non-Hispanic black, Mexican-American), education (less than high school, high school or equivalent, some college, college graduate), marital status (single and never married; married or living with a partner; widowed, divorced, or separated), household size (1, 2, 3–4, 5+), currently participating in a health insurance plan (yes, no), body mass index (less than 18.5, 18.5–24.9, 25–29.9, 30+), income-to-poverty ratio (less than 0.5, 0.51–1.0, 1.01–1.3, greater than 1.3), and whether the dietary recall was completed on a weekday or weekend. Differences in baseline nutrient intake across groups, and differences between baseline intake and each of the modeled intakes within each group were tested using z-scores, with significance at *P* < 0.05 with Bonferroni adjustment for multiple comparisons. Stata 14 was used for data management and SAS 9.4 was used for all analyses. This study was deemed exempt by the University of North Dakota Institutional Review Board.

## Results

### Sociodemographic characteristics

WIC participants were younger (26.3 y, 95% CI: 25.6–26.9 y) than all other groups (Table 1), and food secure non-participants were older (48.0 y, 47.5–48.5 y). All WIC participants were female, and between 50 and 60% of food insecure non-participants, SNAP participants, and food secure non-participants were female. Most individuals in each group were non-Hispanic white, with the greatest proportion in the food secure non-participant group (82.7%, 80.7–84.6%). All groups had a similar distribution of educational attainment except for food secure non-participants, which had higher educational attainment compared to the other groups. Most individuals in each group were married or living with a partner, with the greatest proportion in the WIC participant group (67.8%, 61.9–73.2%) and food secure non-participant group (65.9%, 64.6–67.1%). WIC participants reported the greatest household size (4.3, 4.2–4.5), and food secure non-participants reported the lowest household size (2.9, 2.8–2.9). Food secure non-participants reported the highest prevalence (84.9%, 83.9–85.8%) of currently participating in a health insurance plan, followed by WIC participants (74.6%, 69.0–79.4%), SNAP participants (67.3%, 64.7–70.0%), and food insecure non-participants (58.0%, 55.3–60.6%). Overweight and obese individuals accounted for 67–76% of each group, yet food secure non-participants had the highest proportion of normal weight individuals (31.2%, 30.2–32.2%). Most (87.2%, 86.2–88.1%) food secure non-participants had an income-to-poverty ratio greater than 1.3, compared with 46.9% (43.9–50.0%) of food insecure non-participants, 35.5% (30.0–41.4%) of WIC participants, and 24.7% (21.7–28.1%) of SNAP participants.

**Table 1:** Sociodemographic characteristics of participants in federal food assistance programs, food insecure individuals, and other individuals 2001–2014 (*n* = 34,741)

Characteristic		n	Food insecure, non-participants (n = 3631)		WIC participants (n = 636)		SNAP participants (n = 4020)		Food secure, non-participants (n = 26,454)	
		% (95% CI)^a^								
Age (y)		34,741	41.5	(40.5–42.4)	26.3	(25.6–26.9)	42.9	(42.1–43.7)	48.0	(47.5–48.5)
Female		34,741	50.0	(47.8–51.4)	100.0	(NA)	59.0	(57.1–60.9)	50.7	(50.1–51.3)
Race-ethnicity		29,696								
Non-Hispanic white			56.9	(52.0–61.6)	43.0	(35.4–50.8)	56.7	(50.7–62.4)	82.7	(80.7–84.6)
Non-Hispanic black			20.3	(17.5–23.5)	28.3	(22.6–34.9)	30.6	(26.3–25.4)	10.0	(8.8–11.3)
Mexican American			22.8	(19.3–26.8)	28.7	(22.8–35.4)	12.7	(9.7–16.4)	7.3	(6.1–8.6)
Education		34,704								
Less than high school			33.9	(31.1–36.7)	34.0	(28.8–40.0)	39.5	(37.0–42.1)	13.6	(12.6–14.7)
High school or equivalent			27.0	(24.8–29.2)	34.1	(26.7–36.5)	28.4	(26.1–31.0)	22.9	(21.8–24.0)
Some college			31.4	(28.8–34.2)	29.0	(24.2–34.2)	27.1	(25.1–29.1)	31.8	(30.8–32.7)
College graduate			7.7	(6.4–9.3)	5.6	(3.7–8.5)	5.0	(3.9–6.3)	31.7	(30.1–33.4)
Marital status		34,723								
Single, never married			25.0	(22.3–27.9)	26.6	(21.6–32.1)	29.5	(26.8–32.4)	16.7	(15.5–18.0)
Married or living with partner			50.5	(47.6–53.4)	67.8	(61.9–73.2)	39.7	(37.1–42.5)	65.9	(64.6–67.1)
Widowed, divorced, or separated			24.5	(22.4–26.8)	5.7	(3.5–9.0)	30.7	(28.3–33.2)	17.4	(16.7–18.1)
Household size (n)		34,741	3.3	(3.2–3.4)	4.3	(4.2–4.5)	3.6	(3.46–3.78)	2.9	(2.8–2.9)
Health insurance^b^		34,620	58.0	(55.3–60.6)	74.6	(69.0–79.4)	67.3	(64.7–70.0)	84.9	(83.9–85.8)
Weight status		34,066								
Underweight			2.6	(1.9–3.5)	0.9	(0.3–2.5)	2.7	(2.1–3.6)	1.5	(1.3–1.7)
Normal weight			28.5	(26.2–31.0)	22.9	(18.3–28.2)	25.5	(23.5–27.6)	31.2	(30.2–32.2)
Overweight			30.0	(27.7–32.4)	32.2	(26.7–38.3)	27.7	(25.7–29.8)	34.6	(33.6–35.6)
Obese			38.9	(36.4–41.5)	44.0	(38.6–49.7)	44.1	(41.3–46.8)	32.7	(31.6–33.8)
Income-to-poverty ratio		32,247								
Less than or equal to 0.50			11.3	(9.5–13.3)	19.9	(15.2–25.5)	21.1	(18.9–23.5)	2.3	(1.9–2.7)
0.51 to 1.00			22.0	(19.6–24.6)	30.9	(26.2–36.1)	40.3	(37.0–43.7)	5.1	(4.7–5.6)
1.01 to 1.30			19.9	(17.4–22.5)	13.7	(10.3–18.1)	13.8	(12.5–15.3)	5.4	(4.9–6.0)
> 1.30			46.9	(43.9–50.0)	35.5	(30.0–41.4)	24.7	(21.7–28.1)	87.2	(86.2–88.1)

^a^Does not apply to age or household size

^b^Participated in health insurance plan at time of survey

Some SNAP participants may have income-to-poverty ratios greater than 1.3 because income test are the primary, but not the only, criterion of program eligibility. For example, Income tests are relaxed if all family members participate in Temporary Assistance for Needy Families (TANF) or receive Supplemental Security Income (SSI). Additionally, individuals move in and out of SNAP on a monthly basis whereas income data were collected on an annual basis

### Most commonly consumed dishes at each eating occasion

Among each group, the most commonly consumed main dishes at breakfast, lunch, and dinner were breakfast cereal, sandwiches (including hotdogs and sausages), and poultry dishes (including mixed dishes with poultry and vegetables), respectively (Additional file 3: Table S3). Whole eggs and scrambled eggs were the most commonly consumed egg dishes at breakfast, lunch, and dinner among each group (Additional file 4: Table S4). Mean consumption amounts and nutrient profiles are presented in Additional file 5: Table S5 and Additional file 6: Table S6, respectively.

### Reported nutrient intake

Table 2 displays reported daily nutrient intakes among each group, with food insecure non-participants as the reference group. The percent not meeting recommendations for vitamin C and calcium was lower, and the percent not meeting recommendations for vitamin E was higher, among WIC participants compared to food insecure non-participants (*P* < 0.001 for vitamin C, and *P* < 0.01 for vitamin E and calcium). WIC participants also had higher mean intakes of energy (*P* = 0.023), protein (*P* = 0.009), and carbohydrates (*P* = 0.007) compared to food insecure non-participants. The percent not meeting recommendations for vitamin D among food insecure non-participants (98.0%, 97.8–98.2%) was higher than SNAP participants (96.0%, 95.8–96.2%; *P* < 0.001); and the percent not meeting recommendations for folate (30.9%, 28.4–33.5%) and zinc (47.0%, 44.7–49.3%) among food insecure non-participants was lower than SNAP participants (folate: 35.7%, 33.2–38.1%; *P* < 0.05 and zinc: 52.4%, 49.7–55.2%; *P* < 0.01). Compared to food insecure non-participants, the percent not meeting recommendations was lower among food secure non-participants for all nutrients (*P* < 0.01 or *P* < 0.001 for all comparisons) except for saturated fatty acids (higher, *P* < 0.01), sodium (higher, *P* < 0.001), niacin (no difference, *P* = 0.251), and folate (no difference, *P* = 0.036).

**Table 2:** Reported daily nutrient intakes among federal food assistance program participants, food insecure non-participants, and food secure non-participants, 2001–2014 (n = 34,741)

Nutrient	Food insecure non-participants^a^ (n = 3631)		WIC participants (n = 636)		SNAP participants (n = 4020)		Food secure non-participants (26,454)	
	Percent not meeting recommendations (95% CI)^d^							
Saturated fatty acids^c^	55.3	(52.1–58.5)	57.5	(50.4–64.7)	58.9	(55.5–62.2)	62.1	(60.6–63.6)**
Vitamin A^d^	67.5	(64.7–70.2)	60.9	(53.3–68.6)	66.4	(62.8–70.0)	46.9	(45.4–48.4)***
Vitamin C	58.7	(55.9–61.4)	47.3	(39.2–55.3)***	60.3	(57.4–55.3)	45.9	(44.3–47.5)***
Vitamin D	98.0	(97.8–98.2)	96.0	(94.0–98.0)	96.0	(95.8–96.2)***	95.0	(94.8–95.2)***
Vitamin E	94.9	(93.9–95.8)	97.3	(95.9–98.6)**	95.1	(94.1–96.1)	88.1	(87.0–89.2)***
Thiamin	12.0	(10.5–13.6)	15.8	(12.6–18.9)	11.6	(10.2–13.0)	9.5	(8.8–10.2)***
Riboflavin	12.5	(10.9–14.0)	12.7	(9.8–15.6)	11.6	(10.2–13.0)	9.7	(9.0–10.4)***
Niacin	10.2	(8.8–11.6)	9.6	(6.9–12.3)	9.6	(8.2–10.9)	9.3	(8.5–10.0)
Vitamin B_6_	22.6	(20.5–24.6)	25.7	(20.6–30.9)	23.8	(21.9–25.8)	18.8	(17.6–19.9)***
Folate^e^	30.9	(28.4–33.5)	27.3	(22.3–32.2)	35.7	(33.2–38.1)*	27.9	(26.8–29.0)
Vitamin B_12_	13.6	(12.0–15.3)	11.3	(8.7–13.9)	12.3	(10.7–13.9)	10.8	(10.0–11.6)**
Calcium	54.7	(52.0–57.4)	44.6	(37.8–51.4)**	55.5	(52.7–58.2)	47.4	(46.2–48.6)***
Iron	15.7	(14.1–17.4)	62.6	(50.6–74.6)***	17.2	(15.4–19)	12.7	(11.9–13.5)***
Magnesium	83.1	(81.3–84.9)	86.9	(83.5–90.4)	85.8	(84.1–87.6)	72.1	(70.9–73.3)***
Sodium^f^	78.7	(76.9–80.5)	81.4	(76.6–86.2)	76.5	(74.5–78.5)	81.4	(80.5–82.3)***
Zinc	47.0	(44.7–49.3)	44.2	(38.5–49.9)	52.4	(49.7–55.2)**	39.4	(38.2–40.6)**
	Mean intake (95% CI)							
Energy (kcal)	2171	(2119–2224)	2309	(2202–2416)*	2192	(2144–2239)	2176	(2159–2192)
Protein, total (g)	81	(79–83)	88	(83–92)*	81	(80–83)	84	(83–84)*
Carbohydrate, total (g)	267	(260–274)	289	(275–304)*	270	(263–277)	262	(260–264)
Fatty acids, total (g)	81	(78–83)	87	(81–92)	81	(79–83)	83	(82–84)
Fiber (g)	14	(13–14)	14	(13–15)	13	(12–13)**	16	(16–16)***
Eicosapentaenoic acid (g)	0.02	(0.02–0.02)	0.02	(0.02–0.03)	0.02	(0.02–0.02)	0.03	(0.03–0.03)**
Docosahexaenoic acid (g)	0.07	(0.06–0.08)	0.07	(0.06–0.08)	0.06	(0.06–0.06)**	0.08	(0.08–0.08)*
α-linolenic acid (g)	1.40	(1.35–1.44)	1.38	(1.26–1.5)	1.35	(1.31–1.4)	1.53	(1.5–1.55)***
Vitamin K (μg)	76	(72–79)	72	(65–80)	72	(68–76)	100	(97–103)***
Choline (mg)	291	(282–300)	269	(251–287)	294	(285–302)	317	(312–322)***
Potassium (mg)	2586	(2555–2618)	2252	(2113–2392)	2586	(2555–2618)	2222	(2159–2286)***
α-carotene (μg)	320	(292–349)	352	(274–430)	516	(488–544)***	516	(488–544)***
β-carotene (μg)	1417	(1331–1504)	1395	(1187–1603)	2136	(2054–2218)**	2136	(2054–2218)***
β-cryptoxanthin (μg)	84	(78–90)	102	(81–123)	78	(73–83)	112	(108–116)***
Lutein and zeaxanthin (μg)	998	(938–1058)	1040	(914–1166)	931	(875–987)	1418	(1362–1473)***
Lycopene (μg)	7514	(6764–8264)	7939	(6436–9442)	6184	(5627–6742)**	8218	(7838–8598)

All estimates adjusted for age, gender, race-ethnicity, education, marital status, household size, health insurance, BMI, income-to-poverty ratio, and whether the dietary recall was on a weekday or weekend

^a^Reference group

^b^Daily nutrient recommendations are Estimated Average Requirements (EAR) unless otherwise specified. National Academy of Sciences, Institute of Medicine. 2006. Dietary reference intakes: The essential guide to nutrient requirements. Jennifer J. Otten, Jennifer Pitzi Hellwig, Linda D. Meyers (eds.). National Academies Press, Washington, DC

^c^Recommendation is less than 10% of total energy. US Department of Health and Human Services & US Department of Agriculture. 2015–2020. Dietary Guidelines for Americans 2015–2020. U.S. Government Printing Office, Washington, DC. Available at: http://health.gov/dietaryguidelines/ (verified 29 March 2016)

^d^Retinol Activity Equivalent (RAE)

^e^Dietary Folate Equivalent (DFE)

^6^Tolerable upper intake level limit (UL). National Academy of Sciences, Institute of Medicine. 2006. Dietary reference intakes: The essential guide to nutrient requirements. Jennifer J. Otten, Jennifer Pitzi Hellwig, Linda D. Meyers (eds.). National Academies Press, Washington, DC

*Different than food insecure non-participants at *P* < 0.017 (Bonferroni adjusted for multiple comparisons)

**Different than food insecure non-participants at *P* < 0.003 (Bonferroni adjusted for multiple comparisons)

***Different than food insecure non-participants at *P* < 0.0003 (Bonferroni adjusted for multiple comparisons)

No difference in mean intake was observed between food insecure non-participants and WIC participants (Table 2) for total fatty acids (*P* = 0.045), fiber (*P* = 0.808), α-linolenic acid (*P* = 0.776), vitamin K (*P* = 0.373), choline (*P* = 0.033), potassium (*P* = 0.557), α-carotene (*P* = 0.455), β-carotene (*P* = 0.846), β-cryptoxanthin (*P* = 0.109), lutein and zeaxanthin (*P* = 0.557), and lycopene (*P* = 0.620). Compared to food insecure non-participants, SNAP participants had lower mean intake of fiber and lycopene (*P* < 0.01 for both comparisons), and higher mean intake of α-carotene (*P* < 0.001) and β-carotene (*P* < 0.01). Food secure non-participants had lower mean intake of potassium, and higher mean intake of protein, fiber, α-linolenic acid, vitamin K, choline, α-carotene, β-carotene, β-cryptoxanthin, and lutein and zeaxanthin compared to food insecure non-participants (*P* < 0.001 for all comparisons except protein: *P* = 0.012).

### Modeled nutrient intake: substituting eggs as the main dish

Tables 3, 4, 5 and 6 display the results of models that substituted the most commonly consumed egg dish for the most commonly consumed main dish at each eating occasion among food insecure non-participants **(**Table 3), WIC participants (Table 4), SNAP participants (Table 5), and food secure non-participants (Table 6).

**Table 3:** Reported daily nutrient intakes and modeled nutrient intakes if eggs were consumed as the main dish at each eating occasion among food insecure non-participants, 2001–2014 (*n* = 3631)

Nutrient	Reported daily intake^2,3^		Modeled daily intake^a^					
			Eggs consumed as main dish at breakfast		Eggs consumed as main dish at lunch		Eggs consumed as main dish at dinner	
	Percent not meeting recommendations (95% CI)^d^							
Saturated fatty acids^e^	55.3	(52.1–58.5)	57.4	(54.0–60.7)	44.2	(41.0–47.4)***	59.2	(56.1–62.4)
Vitamin A^f^	67.5	(64.7–70.2)	70.0	(67.4–72.7)	63.3	(60.5–66.2)	61.3	(58.1–64.4)**
Vitamin C	58.7	(55.9–61.4)	59.2	(56.5–61.9)	58.8	(56.0–61.5)	58.9	(56.3–61.6)
Vitamin D	98.0	(97.8–98.2)	98.0	(97.8–98.2)	94.0	(93.8–94.2)**	94.0	(93.8–94.2)**
Vitamin E	94.9	(93.9–95.8)	95.4	(94.4–96.3)	94.1	(93.0–95.1)	94.0	(92.9–95.0)
Thiamin	12.0	(10.5–13.6)	12.3	(10.7–13.8)	11.5	(10.0–13.0)	10.7	(9.2–12.2)
Riboflavin	12.5	(10.9–14.0)	12.8	(11.2–14.3)	11.9	(10.3–13.4)	11.0	(9.5–12.5)
Niacin	10.2	(8.8–11.6)	10.7	(9.3–12.1)	10.6	(9.1–12.1)	10.2	(8.8–11.6)
Vitamin B_6_	22.6	(20.5–24.6)	24.6	(22.6–26.7)	22.7	(20.8–24.7)	24.8	(22.8–26.8)
Folate^g^	30.9	(28.4–33.5)	34.8	(32.1–37.5)	31.0	(28.5–33.6)	29.9	(27.4–32.4)
Vitamin B_12_	13.6	(12.0–15.3)	13.8	(12.1–15.5)	13.4	(11.8–15.0)	12.0	(10.5–13.5)
Calcium	54.7	(52.0–57.4)	55.1	(52.4–57.9)	55.3	(52.6–58.0)	53.6	(50.8–56.3)
Iron	15.7	(14.1–17.4)	15.7	(14.1–17.3)	15.7	(14.1–17.4)	15.3	(13.7–17.0)
Magnesium	83.1	(81.3–84.9)	84.2	(82.4–85.9)	71.8	(69.4–74.2)***	83.7	(82.0–85.4)
Sodium^8^	78.7	(76.9–80.5)	78.5	(76.7–80.2)	77.8	(76.0–79.6)	78.5	(76.7–80.3)
Zinc	47.0	(44.7–49.3)	49.7	(47.3–52.0)	48.0	(45.8–50.3)	47.1	(44.8–49.4)
	Mean intake (95% CI)							
Energy (kcal)	2171	(2119–2224)	2157	(2104–2210)	2162	(2110–2215)	2167	(2114–2220)
Protein, total (g)	81	(79–83)	80	(78–83)	79	(76–81)	75	(73–78)**
Carbohydrate, total (g)	267	(260–274)	262	(255–269)	267	(260–274)	270	(263–277)
Fatty acids, total (g)	81	(78–83)	81	(78–83)	79	(76–81)	78	(76–81)
Fiber (g)	14	(13–14)	14	(13–14)	14	(13–14)	14	(13–14)
Eicosapentaenoic acid (g)	0.02	(0.02–0.02)	0.02	(0.02–0.02)	0.02	(0.02–0.02)	0.02	(0.02–0.02)
Docosahexaenoic acid (g)	0.07	(0.06–0.08)	0.07	(0.06–0.07)	0.07	(0.07–0.08)	0.08	(0.07–0.08)
α-linolenic acid (g)	1.40	(1.35–1.44)	1.40	(1.35–1.44)	1.38	(1.34–1.43)	1.40	(1.35–1.44)
Vitamin K (μg)	76	(72–79)	76	(72–79)	76	(72–79)	75	(72–79)
Choline (mg)	291	(282–300)	302	(292–312)	324	(314–335)***	346	(334–358)***
Potassium (mg)	2586	(2555–2618)	2569	(2539–2599)	2566	(2536–2596)	2561	(2530–2592)
α-carotene (μg)	320	(292–349)	282	(260–304)	283	(198–368)	268	(156–379)
β-carotene (μg)	1417	(1331–1504)	1418	(1333–1504)	1411	(1326–1496)	1389	(1306–1472)
β-cryptoxanthin (μg)	84	(78–90)	77	(72–82)	82	(76–87)	80	(75–85)
Lutein and zeaxanthin (μg)	998	(938–1058)	996	(936–1055)	1064	(1004–1125)	1110	(1046–1175)
Lycopene (μg)	7514	(6764–8264)	7403	(6717–8088)	7775	(7005–8546)	7578	(6818–8338)

All estimates adjusted for age, gender, race-ethnicity, education, marital status, household size, health insurance, BMI, income-to-poverty ratio, and whether the dietary recall was on a weekday or weekend

^a^Modeled daily nutrient intake if eggs were consumed as the main dish at each eating occasion

^b^Mean, 2001–2014

^c^Referent

^d^Daily nutrient recommendations are Estimated Average Requirements (EAR) unless otherwise specified. National Academy of Sciences, Institute of Medicine. 2006. Dietary reference intakes: The essential guide to nutrient requirements. Jennifer J. Otten, Jennifer Pitzi Hellwig, Linda D. Meyers (eds.). National Academies Press, Washington, DC

^e^Less than 10% of total energy. US Department of Health and Human Services & US Department of Agriculture. 2015–2020. Dietary Guidelines for Americans 2015–2020. U.S. Government Printing Office, Washington, DC. Available at: http://health.gov/dietaryguidelines/ (verified 29 March 2016)

^f^Retinol Activity Equivalent (RAE)

^g^Dietary Folate Equivalent (DFE)

^h^Tolerable upper intake level limit. National Academy of Sciences, Institute of Medicine. 2006. Dietary reference intakes: The essential guide to nutrient requirements. Jennifer J. Otten, Jennifer Pitzi Hellwig, Linda D. Meyers (eds.). National Academies Press, Washington, DC

*Different than food insecure non-participants at *P* < 0.017 (Bonferroni adjusted for multiple comparisons)

**Different than food insecure non-participants at *P* < 0.003 (Bonferroni adjusted for multiple comparisons)

**Table 4:** Reported daily nutrient intakes and modeled nutrient intakes if eggs were consumed as the main dish at each eating occasion among participants in the Special Supplemental Nutrition Program for Women, Infants, and Children (WIC), 2001–2014 (*n* = 636)

Nutrient	Reported daily intake^b,c^		Modeled daily intake^a^					
			Eggs consumed as main dish at breakfast		Eggs consumed as main dish at lunch		Eggs consumed as main dish at dinner	
	Percent not meeting recommendations (95% CI)^d^							
Saturated fatty acids^e^	57.5	(50.4–64.7)	62.1	(55.5–68.7)	40.9	(34.1–47.7)**	63.1	(56.5–69.7)
Vitamin A^f^	60.9	(53.3–68.6)	66.3	(58.8–73.8)	54.5	(47.7–61.2)	54.6	(46.8–62.5)
Vitamin C	47.3	(39.2–55.3)	48.9	(40.8–57.0)	48.9	(40.8–57.0)	47.8	(39.9–55.8)
Vitamin D	96.0	(94.0–98.0)	97.0	(96.8–97.2)	95.0	(94.0–98.0)	96.0	(95.8–96.2)
Vitamin E	97.3	(95.9–98.6)	97.6	(96.5–98.8)	96.7	(95.2–98.3)	96.9	(95.4–98.3)
Thiamin	15.8	(12.6–18.9)	19.0	(15.3–22.6)	15.4	(12.3–18.4)	14.8	(11.6–18.1)
Riboflavin	12.7	(9.8–15.6)	14.3	(11.1–17.4)	11.6	(8.9–14.4)	11.1	(8.3–13.8)
Niacin	9.6	(6.9–12.3)	11.6	(8.4–14.7)	10.9	(7.9–13.8)	9.7	(7.0–12.4)
Vitamin B_6_	25.7	(20.6–30.9)	33.6	(27.3–39.8)	26.4	(21.2–31.6)	29.4	(23.9–34.9)
Folate^j^	27.3	(22.3–32.2)	38.9	(31.8–46.1)**	28.0	(22.9–33.1)	26.6	(21.6–31.6)
Vitamin B_12_	11.3	(8.7–13.9)	12.2	(9.4–15.1)	10.9	(8.4–13.4)	10.0	(7.6–12.5)
Calcium	44.6	(37.8–51.4)	44.92	(38.3–51.6)	45.49	(38.3–52.7)	42.4	(35.8–49.0)
Iron	62.6	(50.6–74.6)	67.4	(56.0–78.9)	62.1	(50.1–74.2)	61.9	(49.8–73.9)
Magnesium	86.9	(83.5–90.4)	88.7	(85.3–92.0)	63.3	(57.1–69.4)***	87.5	(84.1–90.9)
Sodium^h^	81.4	(76.6–86.2)	80.7	(75.6–85.8)	80.9	(75.9–85.9)	81.2	(76.4–85.9)
Zinc	44.2	(38.5–49.9)	51.2	(45.1–57.2)	46.3	(40.1–52.4)	45.5	(39.5–51.5)
	Mean intake (95% CI)							
Energy (kcal)	2309	(2202–2416)	2279	(2172–2387)	2300	(2193–2408)	2304	(2198–2410)
Protein, total (g)	88	(83–92)	88	(83–92)	85	(80–89)	78	(77–79)**
Carbohydrate, total (g)	289	(275–304)	256	(254–258)**	289	(275–304)	298	(283–313)
Fatty acids, total (g)	87	(81–92)	88	(82–93)	85	(79–90)	88	(82–93)
Fiber (g)	14	(13–15)	13	(13–14)	14	(13–15)	14	(13–15)
Eicosapentaenoic acid (g)	0.02	(0.02–0.03)	0.02	(0.02–0.03)	0.023	(0.02–0.02)	0.019	(0.02–0.02)
Docosahexaenoic acid (g)	0.07	(0.06–0.08)	0.07	(0.06–0.08)	0.079	(0.06–0.08)	0.069	(0.06–0.08)
α-linolenic acid (g)	1.38	(1.26–1.5)	1.40	(1.28–1.51)	1.36	(1.24–1.48)	1.41	(1.29–1.52)
Vitamin K (μg)	72	(65–80)	72	(65–80)	71	(63–78)	71	(64–79)
Choline (mg)	269	(251–287)	286	(267–305)	316	(294–337)**	308	(285–331)***
Potassium (mg)	2252	(2113–2392)	2233	(2092–2373)	2215	(2070–2360)	2222	(2083–2362)
α-carotene (μg)	352	(274–430)	309	(252–367)	307	(200–414)	314	(174–453)
β-carotene (μg)	1395	(1187–1603)	1386	(1176–1596)	1368	(1158–1578)	1390	(1181–1599)
β-cryptoxanthin (μg)	102	(81–123)	92	(75–110)	100	(82–118)	96	(80–113)
Lutein and zeaxanthin (μg)	1040	(914–1166)	1034	(916–1151)	1158	(1038–1279)	1141	(1013–1268)
Lycopene (μg)	7939	(6436–9442)	8249	(6743–9756)	8078	(6402–9754)	9180	(7504–10,857)

All estimates adjusted for age, race-ethnicity, education, marital status, household size, health insurance, BMI, income-to-poverty ratio, and whether the dietary recall was on a weekday or weekend

^a^Modeled daily nutrient intake if eggs were consumed as the main dish at each eating occasion

^b^Mean, 2001–2014

^3^Referent

^d^Daily nutrient recommendations are Estimated Average Requirements (EAR) unless otherwise specified. National Academy of Sciences, Institute of Medicine. 2006. Dietary reference intakes: The essential guide to nutrient requirements. Jennifer J. Otten, Jennifer Pitzi Hellwig, Linda D. Meyers (eds.). National Academies Press, Washington, DC

^e^Less than 10% of total energy. US Department of Health and Human Services & US Department of Agriculture. 2015–2020. Dietary Guidelines for Americans 2015–2020. U.S. Government Printing Office, Washington, DC. Available at: http://health.gov/dietaryguidelines/ (verified 29 March 2016)

^f^Retinol Activity Equivalent (RAE)

^j^Dietary Folate Equivalent (DFE)

^h^Tolerable upper intake level limit. National Academy of Sciences, Institute of Medicine. 2006. Dietary reference intakes: The essential guide to nutrient requirements. Jennifer J. Otten, Jennifer Pitzi Hellwig, Linda D. Meyers (eds.). National Academies Press, Washington, DC

*Different than food insecure non-participants at *P* < 0.017 (Bonferroni adjusted for multiple comparisons)

**Different than food insecure non-participants at *P* < 0.003 (Bonferroni adjusted for multiple comparisons)

***Different than food insecure non-participants at *P* < 0.0003 (Bonferroni adjusted for multiple comparisons)

**Table 5:** Reported daily nutrient intakes and modeled nutrient intakes if eggs were consumed as the main dish at each eating occasion among participants in the Supplemental Nutrition Assistance Program (SNAP), 2001–2014 (*n* = 4020)

Nutrient	Reported daily intake^b,c^		Modeled daily intake^a^					
			Eggs consumed as main dish at breakfast		Eggs consumed as main dish at lunch		Eggs consumed as main dish at dinner	
	Percent not meeting recommendations (95% CI)^d^							
Saturated fatty acids^e^	58.9	(55.5–62.2)	61.9	(58.5–65.3)	40.1	(36.8–43.4)***	63.5	(60.2–66.8)
Vitamin A^f^	66.4	(62.8–70.0)	70.1	(66.7–73.6)	62.5	(58.8–66.3)	59.4	(55.7–63.1)**
Vitamin C	60.3	(57.4–55.3)	61.4	(58.6–64.2)	60.0	(57.3–62.7)	60.4	(57.6–63.1)
Vitamin D	96.0	(95.8–96.2)	97.0	(96.8–97.2)**	96.0	(95.8–96.2)	95.0	(94.8–95.2)**
Vitamin E	95.1	(94.1–96.1)	95.6	(94.7–96.5)	94.6	(93.5–95.6)	94.3	(93.2–95.4)
Thiamin	11.6	(10.2–13.0)	12.3	(10.9–13.8)	11.1	(9.6–12.5)	10.1	(8.7–11.4)
Riboflavin	12.0	(10.6–13.5)	12.6	(11.1–14.1)	11.4	(9.9–12.8)	10.1	(8.7–11.4)
Niacin	9.6	(8.2–10.9)	10.6	(9.2–12.0)	10.1	(8.7–11.4)	9.5	(8.1–10.9)
Vitamin B_6_	23.8	(21.9–25.8)	28.3	(26.1–30.4)**	24.6	(22.6–26.5)	27.1	(25.0–29.1)
Folate^g^	35.7	(33.2–38.1)	43.4	(41.0–45.8)**	36.0	(33.5–38.6)	34.4	(31.8–36.9)
Vitamin B_12_	12.3	(10.7–13.9)	13.0	(11.3–14.6)	12.1	(10.5–13.8)	10.6	(9.1–12.1)
Calcium	55.5	(52.7–58.2)	55.61	(52.8–58.4)	55.18	(52.5–57.9)	54.2	(51.5–56.9)
Iron	17.2	(15.4–19)	18.71	(16.9–20.5)	17.44	(15.6–19.3)	16.5	(14.7–18.3)
Magnesium	85.8	(84.1–87.6)	87.1	(85.4–88.9)	74.1	(71.6–76.6)***	86.4	(84.7–88.0)
Sodium^h^	76.5	(74.5–78.5)	76.2	(74.2–78.2)	75.2	(73.2–77.3)	76.1	(74.1–78.2)
Zinc	52.4	(49.7–55.2)	56.5	(53.7–59.2)	53.5	(50.8–56.3)	52.6	(49.9–55.2)
	Mean intake (95% CI)							
Energy (kcal)	2192	(2144–2239)	2173	(2125–2220)	2183	(2135–2230)	2183	(2136–2231)
Protein, total (g)	81	(80–83)	81	(80–83)	81	(79–82)	84	(80–88)
Carbohydrate, total (g)	270	(263–277)	281	(266–296)	273	(266–280)	272	(265–279)
Fatty acids, total (g)	81	(79–83)	82	(80–84)	82	(80–84)	79	(77–81)
Fiber (g)	13	(12–13)	12	(12–13)	13	(12–14)	13	(12–13)
Eicosapentaenoic acid (g)	0.02	(0.02–0.02)	0.02	(0.02–0.02)	0.02	(0.02–0.02)	0.021	(0.02–0.02)
Docosahexaenoic acid (g)	0.06	(0.06–0.06)	0.06	(0.05–0.06)	0.06	(0.06–0.07)	0.071	(0.07–0.08)
α-linolenic acid (g)	1.35	(1.31–1.4)	1.36	(1.32–1.41)	1.35	(1.31–1.4)	1.35	(1.3–1.39)
Vitamin K (μg)	72	(68–76)	72	(69–76)	72	(68–76)	71	(68–75)
Choline (mg)	294	(285–302)	304	(296–313)	322	(312–331)***	351	(340–361)***
Potassium (mg)	2586	(2555–2618)	2569	(2539–2599)	2566	(2536–2596)	2561	(2530–2592)
α-carotene (μg)	246	(218–274)	227	(204–250)	226	(157–295)	214	(124–305)
β-carotene (μg)	1231	(1133–1329)	1234	(1139–1329)	1241	(1142–1339)	1213	(1120–1306)
β-cryptoxanthin (μg)	78	(73–83)	72	(68–77)	75	(70–79)	76	(71–81)
Lutein and zeaxanthin (μg)	931	(875–987)	925	(871–979)	986	(928–1044)	1053	(995–1111)**
Lycopene (μg)	6184	(5627–6742)	6386	(5811–6961)	6833	(6206–7461)	6274	(5693–6855)

All estimates adjusted for age, gender, race-ethnicity, education, marital status, household size, health insurance, BMI, income-to-poverty ratio, and whether the dietary recall was on a weekday or weekend

^a^Modeled daily nutrient intake if eggs were consumed as the main dish at each eating occasion

^b^Mean, 2001–2014

^c^Referent

^d^Daily nutrient recommendations are Estimated Average Requirements (EAR) unless otherwise specified. National Academy of Sciences, Institute of Medicine. 2006. Dietary reference intakes: The essential guide to nutrient requirements. Jennifer J. Otten, Jennifer Pitzi Hellwig, Linda D. Meyers (eds.). National Academies Press, Washington, DC

^e^Less than 10% of total energy. US Department of Health and Human Services & US Department of Agriculture. 2015–2020. Dietary Guidelines for Americans 2015–2020. U.S. Government Printing Office, Washington, DC. Available at: http://health.gov/dietaryguidelines/ (verified 29 March 2016)

^f^Retinol Activity Equivalent (RAE)

^f^Dietary Folate Equivalent (DFE)

^j^Tolerable upper intake level limit. National Academy of Sciences, Institute of Medicine. 2006. Dietary reference intakes: The essential guide to nutrient requirements. Jennifer J. Otten, Jennifer Pitzi Hellwig, Linda D. Meyers (eds.). National Academies Press, Washington, DC

*Different than food insecure non-participants at *P* < 0.017 (Bonferroni adjusted for multiple comparisons)

**Different than food insecure non-participants at *P* < 0.003 (Bonferroni adjusted for multiple comparisons)

***Different than food insecure non-participants at *P* < 0.0003 (Bonferroni adjusted for multiple comparisons)

**Table 6:** Reported daily nutrient intakes and modeled nutrient intakes if eggs were consumed as the main dish at each eating occasion among food secure non-participants, 2001–2014 (*n* = 26,454)

Nutrient	Reported daily intake^b,c^		Modeled daily intake^a^					
			Eggs consumed as main dish at breakfast		Eggs consumed as main dish at lunch		Eggs consumed as main dish at dinner	
	Percent not meeting recommendations (95% CI)^d^							
Saturated fatty acids^e^	62.1	(60.6–63.6)	65.6	(64.2–67.1)*	37.0	(35.4–38.5)***	66.3	(64.8–67.8)**
Vitamin A^f^	46.9	(45.4–48.4)	50.1	(48.6–51.6)**	42.7	(41.2–44.2)***	40.4	(38.9–41.9)**
Vitamin C	45.9	(44.3–47.5)	47.2	(45.6–48.8)	46.1	(44.5–47.7)	46.4	(44.8–48.0)
Vitamin D	95.0	(94.8–95.2)	95.0	(94.8–95.2)	94.0	(93.8–94.2)**	94.0	(93.8–94.2)**
Vitamin E	88.1	(87.0–89.2)	89.7	(88.7–90.6)	87.0	(85.9–88.1)	86.5	(85.4–87.6)
Thiamin	9.5	(8.8–10.2)	9.7	(9.0–10.5)	9.3	(8.5–10.0)	8.9	(8.1–9.6)
Riboflavin	9.7	(9.0–10.4)	9.9	(9.2–10.7)	9.4	(8.6–10.1)	8.9	(8.2–9.6)
Niacin	9.3	(8.5–10.0)	10.2	(9.4–10.9)	9.6	(8.8–10.3)	9.3	(8.5–10.0)
Vitamin B_6_	18.8	(17.6–19.9)	22.6	(21.3–23.9)***	19.1	(18.0–20.3)	20.8	(19.6–21.9)
Folate^j^	27.9	(26.8–29.0)	35.8	(34.6–37.0)**	27.9	(26.7–29.0)	27.1	(25.9–28.2)
Vitamin B_12_	10.8	(10.0–11.6)	11.4	(10.5–12.3)	10.6	(9.8–11.4)	9.9	(9.1–10.6)
Calcium	47.4	(46.2–48.6)	48.32	(47.1–49.5)	47.55	(46.4–48.7)	46.3	(45.1–47.5)
Iron	12.7	(11.9–13.5)	13.62	(12.8–14.4)	12.61	(11.8–13.4)	12.3	(11.6–13.1)
Magnesium	72.1	(70.9–73.3)	74.7	(73.5–75.8)*	58.8	(57.3–60.3)***	73.1	(71.9–74.3)
Sodium^h^	81.4	(80.5–82.3)	81.1	(80.2–82.0)	80.5	(79.6–81.4)	81.3	(80.5–82.2)
Zinc	39.4	(38.2–40.6)	44.0	(42.8–45.3)***	40.1	(38.9–41.3)	39.5	(38.2–40.7)
	Mean intake (95% CI)							
Energy (kcal)	2176	(2159–2192)	2158	(2142–2175)	2170	(2153–2186)	2171	(2154–2187)
Protein, total (g)	84	(83–84)	83	(82–84)	81	(81–82)***	76	(74–78)***
Carbohydrate, total (g)	262	(260–264)	264	(258–271)	262	(260–264)	265	(262–267)
Fatty acids, total (g)	83	(82–84)	83	(82–83)	81	(80–82)*	80	(79–81)
Fiber (g)	16	(16–16)	15	(15–15)***	16	(15–16)	16	(15–16)
Eicosapentaenoic acid (g)	0.03	(0.03–0.03)	0.03	(0.03–0.03)	0.03	(0.02–0.03)	0.03	(0.02–0.03)
Docosahexaenoic acid (g)	0.08	(0.08–0.08)	0.08	(0.08–0.08)	0.08	(0.08–0.09)	0.09	(0.09–0.09)
α-linolenic acid (g)	1.53	(1.50–1.55)	1.53	(1.51–1.55)	1.52	(1.50–1.54)	1.53	(1.50–1.55)
Vitamin K (μg)	100	(97–103)	100	(97–103)	100	(97–103)	99	(96–102)
Choline (mg)	317	(312–322)	336	(331–341)	353	(348–358)***	382	(376–388)***
Potassium (mg)	2222	(2159–2286)	2219	(2158–2279)	2207	(2145–2269)	2205	(2143–2267)
α-carotene (μg)	516	(488–544)	426	(405–446)	425	(300–551)	403	(236–570)
β-carotene (μg)	2136	(2054–2218)	2134	(2053–2214)	2138	(2057–2219)	2104	(2022–2186)
β-cryptoxanthin (μg)	112	(108–116)	103	(99–107)	107	(103–111)	105	(101–109)
Lutein and zeaxanthin (μg)	1418	(1362–1473)	1436	(1383–1490)	1489	(1435–1543)	1544	(1491–1598)**
Lycopene (μg)	8218	(7838–8598)	8096	(7759–8433)	8653	(8242–9065)	8290	(7920–8660)

All estimates adjusted for age, gender, race-ethnicity, education, marital status, household size, health insurance, BMI, income-to-poverty ratio, and whether the dietary recall was on a weekday or weekend

^a^Modeled daily nutrient intake if eggs were consumed as the main dish at each eating occasion

^b^Mean, 2001–2014

^c^Referent

^d^Daily nutrient recommendations are Estimated Average Requirements (EAR) unless otherwise specified. National Academy of Sciences, Institute of Medicine. 2006. Dietary reference intakes: The essential guide to nutrient requirements. Jennifer J. Otten, Jennifer Pitzi Hellwig, Linda D. Meyers (eds.). National Academies Press, Washington, DC

^e^Less than 10% of total energy. US Department of Health and Human Services & US Department of Agriculture. 2015–2020. Dietary Guidelines for Americans 2015–2020. U.S. Government Printing Office, Washington, DC. Available at: http://health.gov/dietaryguidelines/ (verified 29 March 2016)

^f^Retinol Activity Equivalent (RAE)

^j^Dietary Folate Equivalent (DFE)

^h^Tolerable upper intake level limit. National Academy of Sciences, Institute of Medicine. 2006. Dietary reference intakes: The essential guide to nutrient requirements. Jennifer J. Otten, Jennifer Pitzi Hellwig, Linda D. Meyers (eds.). National Academies Press, Washington, DC

*Different than food insecure non-participants at *P* < 0.017 (Bonferroni adjusted for multiple comparisons)

**Different than food insecure non-participants at *P* < 0.003 (Bonferroni adjusted for multiple comparisons)

***Different than food insecure non-participants at *P* < 0.0003 (Bonferroni adjusted for multiple comparisons)

Among food insecure non-participants, no changes in the percent not meeting recommended nutrient intakes were observed when eggs were substituted at breakfast (Table 3). At lunch, substituting eggs decreased the percent not meeting daily recommendations for saturated fatty acids (*P* < 0.001), vitamin D (*P* < 0.01), and magnesium (*P* < 0.001). When eggs were substituted as the main dish at dinner, the percent not meeting recommendations decreased for vitamin A (*P* < 0.01) and vitamin D (*P* < 0.01). Among WIC participants (Table 4), substituting eggs at breakfast increased the percent not meeting folate recommendations (*P* < 0.01); at lunch, the percent not meeting recommendations decreased for saturated fatty acids (*P* < 0.01) and magnesium (*P* < 0.001), and mean intake of choline increased (*P* < 0.001). No changes in nutrient intake were observed when eggs were substituted at dinner, except a decrease in mean intake of protein (*P* < 0.001) and an increase in mean intake of choline (*P* < 0.01). Among SNAP participants (Table 5), substituting eggs at breakfast increased the percent not meeting recommendations for vitamin D (*P* < 0.01), vitamin B_6_ (*P* < 0.01), and folate (*P* < 0.01), and substituting eggs at lunch decreased the percent not meeting recommendations for saturated fatty acids (*P* < 0.001) and magnesium (*P* < 0.001). Substituting eggs at dinner decreased the percent not meeting recommendations for vitamin A (*P* < 0.01) and vitamin D (*P* < 0.01). Among food secure non-participants (Table 6), substituting eggs at breakfast increased the percent of individuals not meeting recommendations for saturated fatty acids (*P* < 0.05), vitamin A (*P* < 0.01), vitamin B_6_ (*P* < 0.001), folate (*P* < 0.01), magnesium (*P* < 0.05), and zinc (*P* < 0.001), and decreased the mean intake of fiber (P < 0.001). Substituting eggs at lunch decreased the percent not meeting recommendations for saturated fatty acids (*P* < 0.001), vitamin A (*P* < 0.001), vitamin D (*P* < 0.01), and magnesium (*P* < 0.001), and decreased the mean intake of protein and total fatty acids (P < 0.001). Substituting eggs at dinner increase the percent not meeting recommendations for saturated fatty acids (*P* < 0.001), but decreased the percent not meeting recommendations for vitamins A and D (*P* < 0.01).

### Total nutrient index

**Fig. 1:**
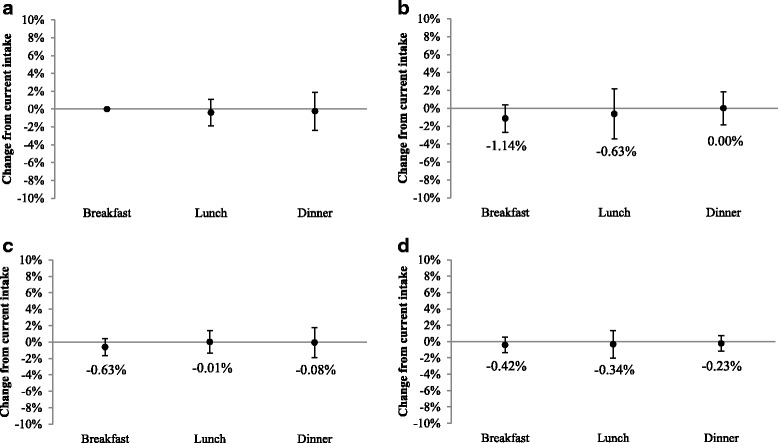
Percent change from reported daily intake of all nutrients if eggs were consumed as the main dish at each eating occasion among **a**) food insecure non-participants (*n* = 3631), **b**) WIC participants (*n* = 636), **c**) SNAP participants (*n* = 4020), and **d**) food secure non-participants (*n* = 26,454), 2001–2014. Adjusted for age, gender, race-ethnicity, education, marital status, household size, currently participating in a health insurance plan, body mass index, income-to-poverty ratio, and whether the dietary recall was on a weekday or weekend

Figure 1a-d display the results of the total nutrient index, which represents the percent change from the reported intake of all 31 nutrients collectively when eggs are substituted for the most commonly consumed main dish at each eating occasion. Among each group (i.e., food insecure non-participants, SNAP participants, WIC participants, and food secure non-participants), total nutrient intake did not change at breakfast, lunch, or dinner (*P* > 0.05). Sensitivity analyses demonstrated that individual nutrients have minimal effect on the reliability of the total nutrient index (Additional file 7: Table S7).

## Discussion

In this nationally representative sample of nearly 35,000 adults surveyed over 14 years, meaningful disparities in daily intake of several key nutrients were observed between distinct populations differentiated by food security status and participation in federal food assistance programs. Importantly, using dietary modeling techniques, we demonstrated that well-defined, moderate dietary changes at the population-level can increase intake of some, but not all, nutrients, and can reduce the prevalence of nutrient inadequacy among these nutritionally vulnerable populations.

Diet modeling has become a popular tool for estimating the impact of food substitutions on nutrient adequacy at the population level [18, 19, 23, 38]. Yet to the best of our knowledge, the present study is the first to use diet modeling to examine the effects of food substitutions at multiple eating occasions on meeting daily nutrient intake recommendations among distinct groups of nutritionally vulnerable populations.

Consistent with our findings, a review by Andreyeva et al. [9] indicated lower nutrient intake among SNAP participants compared to higher income non-participants. Leung et al. [39] analyzed NHANES (1998–2008) dietary data from 918 SNAP participants and reported that 52 and 67% did not meet recommendations for folate and calcium, respectively, which are higher than our estimates (36% for folate and 56% for calcium). These disagreements may stem from differences in sample size (*n* = 3724 SNAP participants in the present study) resulting from different inclusion criteria. In contrast to Leung et al., [39] we categorized SNAP participants regardless of age or income, which allowed for greater sample size and improved generalizability to the target population. To the best of our knowledge, no previous studies have examined nutrient adequacy among adult WIC participants, although several studies have observed an overall improvement in the healthfulness of food choices among WIC participants since 2009 [8, 40]. Our results for the proportion of food insecure non-participants meeting individual nutrient recommendations are largely consistent with others [41].

In the present study, we demonstrated that consuming eggs as the main dish at lunch or dinner did not have an effect on total nutrient intake among nutritionally vulnerable populations (i.e., food insecure non-participants, WIC participants, and SNAP participants) or their food secure non-participant counterparts. However, our findings for several specific nutrients warrant particular mention. Importantly, we observed a meaningful reduction in the prevalence of food insecure non-participants not meeting daily vitamin D recommendations when eggs were consumed as the main dish at lunch or dinner. The 2015–2020 Dietary Guidelines for Americans proclaimed vitamin D a nutrient of public health concern [42] given the strikingly high prevalence of intake inadequacy (92–97%) [43] and its relationship to chronic disease patterns [42]. Yet, when eggs were consumed as the main dish at breakfast, the prevalence of folate inadequacy increased for WIC participants and SNAP participants, which is concerning. Folate is a critical nutrient for fetal neural tube development, which is the reason for elevated intake recommendations during pregnancy [44]. This is of particular concern for adult WIC participants, over 40% of whom are pregnant and, of those, over half enrolled during their first trimester [4] when adequate folate intake is most critical [45]. We estimated that breakfast cereal, much of which is fortified with folic acid, was the most commonly consumed main dish at breakfast among all groups, so it is not surprising that consuming eggs instead of breakfast cereal resulted in reduced folate (i.e. dietary folate equivalent) intake. Although others have recently reported successful innovations in the development of folate-enriched eggs, [46] more research is needed before these products can be successfully introduced to consumers on a large scale.

Several important strengths of this study warrant mention. To the best of our knowledge this is the first study to assess nutrient intake among distinct nutritionally vulnerable populations simultaneously, for a diverse array of 31 nutrients including macronutrients, vitamins, minerals, and carotenoids. NCI methods and macros were used to estimate usual intake distributions, which represent the gold standard for addressing the foremost statistical challenges when assessing nutrient intake among populations [47]. We also used a novel approach, the total nutrient index, to assess intake of all 31 nutrients collectively, which accounts for relative differences in the standard errors of each nutrient. Finally, and importantly, the large sample size (*n* > 34,000) and national representation of our data make our findings generalizable to each of our study populations.

This study is also not without limitations. We conducted our food substitution analyses on a gram basis, ensuring that equal grams of dishes were being substituted; other approaches include using a serving basis or calorie basis. In the present study our data represented main dishes as they were reported consumed, which included a combination of individual foods with distinct serving sizes, thus it was not possible to use the serving basis for this analysis. We tested using the calorie basis for food substitutions and observed that it provided essentially the same results as the gram basis (~ 1% difference in calories between food substitutions). It is also possible that pooling data across NHANES waves may have masked changes in nutrient intake among some groups, particularly WIC participants [8, 40]. Yet it was necessary to pool these data, especially for WIC participants, due to small sample sizes in individual waves, in order to observe clinically and statistically meaningful results.

Regular assessment of nutrient inadequacy at the population level is a crucial component of the public health nutrition agenda in the US, and nutritional monitoring of low income and other vulnerable populations is of particular importance. On the one hand, it is reassuring that populations with a high prevalence of nutrient inadequacy for key nutrients are participating in federal food assistance programs. On the other hand, more research is needed to better understand why many income-eligible food insecure individuals do not participate in these vital programs (over 50% in this study), given their high prevalence of nutrient inadequacy. Continued efforts are needed to ensure that nutritionally vulnerable populations have access to healthy foods, and that at-risk individuals are counseled to make food choices that promote nutrient adequacy. Additional research is needed to better understand how food substitutions affect diet costs, which may be an important driver of food purchasing decisions among low income individuals with limited food budgets [6, 7].

## Conclusions

This study is the first to use diet modeling to examine the effects of food substitutions at multiple eating occasions on meeting daily nutrient intake recommendations among distinct groups of nutritionally vulnerable populations. A novel total nutrient index was also used to estimate changes in the intake of a broad array of diverse nutrients collectively. Regular assessment of nutrient inadequacy at the population level is a crucial component of the public health nutrition agenda in the US, and nutritional monitoring of low income and other vulnerable populations is of particular importance. Clinically meaningful disparities in nutrient intake remain between distinct populations differentiated by food security status and participation in federal food assistance programs. Well-defined and moderate dietary changes at the population-level have the potential to increase nutrient intake and reduce the prevalence of nutrient inadequacy among these nutritionally vulnerable populations, particularly at lunch and dinner. However, careful consideration should be awarded to food substitutions at breakfast, particularly among individuals who are pregnant or trying to become pregnant, in order to maintain adequate folate status. Further research is needed in two key areas: to better understand why many income-eligible food insecure individuals do not participate in federal food assistance programs, and to better understand how food substitutions affect diet costs among low income individuals with limited food budgets.

## Additional files

Additional file 1: **Table S1.** Foods included in main dish groups. (DOCX 21 kb)

Additional file 2: **Table S2.** Daily nutrient intake recommendations. (DOCX 26 kb)

Additional file 3: **Table S3.** Percent of individuals consuming types of main dishes at each eating occasion, 2001–2014 (*n* = 34,741). (DOCX 23 kb)

Additional file 4: **Table S4.** Percent of individuals consuming types of egg dishes at each eating occasion, 2001–2014 (n = 34,741). (DOCX 17 kb)

Additional file 5: **Table S5.** Amount of each dish used in substitution modeling. (DOCX 21 kb)

Additional file 6: **Table S6.** Nutrient content of dishes used in substitution modeling. (DOCX 32 kb)

Additional file 7: **Table S7.** Sensitivity analysis for total nutrient index. (DOCX 27 kb)

## Abbreviations

24HR: 24-h recall
DGA: Dietary guidelines for Americans
FNDDS: Food and nutrient database for dietary studies
NCI: National Cancer Institute
NHANES: National Health and Nutrition Examination Survey
SNAP: Supplemental Nutrition Assistance Program
USDA: United States Department of Agriculture
WIC: Special Supplemental Nutrition Program for Women, Infants, and Children
WWEIA: What we eat in America
