# Supplementary material for: Nutrient intake disparities in the US: modeling the effect of food substitutions
Source: Nutr J. 2018 May 17;17:53. doi: 10.1186/s12937-018-0360-z (PMC5960152; doi:10.1186/s12937-018-0360-z)
Supplement: Supplementary file 1 — Table S1. Foods included in main dish groups. (DOCX 21 kb) [file 12937_2018_360_MOESM1_ESM.docx]

| Supplemental Table 1: Foods included in main dish groups | | | |
| --- | --- | --- | --- |
|  |  |  |  |
| Main dish category | |  | FNDDS code^1^ |
| Beef dish | |  |  |
|  | Beef with starch item |  | 2721 |
|  | Beef with starch and vegetable |  | 2731 |
|  | Beef with vegetable, no potatoes |  | 2741 |
|  | Frozen or shelf-stable beef meals |  | 2811 |
|  | Beef with gravy or sauce |  | 2711 |
|  | Steak |  | 211 |
|  | Oxtails, neckbones, short ribs, head |  | 213 |
|  | Roasts, stew meat, corned beef, brisket | | 214 |
|  | Ground beef |  | 215 |
| Pork dish | |  |  |
|  | Pork with starch item |  | 2722 |
|  | Pork with starch and vegetable |  | 2732 |
|  | Pork with vegetable, no potatoes |  | 2742 |
|  | Pork with gravy or sauce |  | 2712 |
|  | Chops |  | 221 |
|  | Steaks, cutlets |  | 222 |
|  | Ham |  | 223 |
|  | Roasts |  | 224 |
|  | Spareribs, cracklings, skin, misc. parts |  | 227 |
| Bacon | |  |  |
|  | Canadian bacon |  | 225 |
|  | Bacon, salt pork |  | 226 |
|  | Turkey bacon |  | 24208500 and 24208510 |
| Poultry dish | |  |  |
|  | Poultry with starch item |  | 2724 |
|  | Poultry with starch and vegetable |  | 2734 |
|  | Poultry with vegetable, no potatoes |  | 2744 |
|  | Chicken |  | 241 |
|  | Poultry with gravy or sauce |  | 2714 |
|  | Frozen or shelf-stable poultry meals |  | 2814 |
|  | Turkey |  | 242 (except 24208500 and 24208510) |
|  | Duck |  | 243 |
|  | Other poultry |  | 244 |
| Other meat dish | |  |  |
|  | Lamb, veal, or game with starch item |  | 2732 |
|  | Lamb, veal, or game with starch and vegetable | | 2733 |
|  | Lamb, veal, or game with vegetable, no potatoes | | 2743 |
|  | Lamb and goat |  |  |
|  | Lamb, NFS |  | 230 |
|  | Lamb and goat |  | 231 |
|  | Veal |  | 232 |
|  | Frozen or shelf-stable veal meals |  | 2813 |
|  | Game |  | 233 |
|  | Lamb and veal with gravy or sauce |  | 2713 |
| Seafood dish | |  |  |
|  | Seafood |  | 26 |
|  | Seafood with gravy or sauce |  | 2715 |
|  | Frozen seafood meals |  | 2815 |
|  | Seafood with starch item |  | 2725 |
|  | Seafood with starch and vegetable |  | 2735 |
|  | Seafood with vegetable, no potatoes |  | 2745 |
| Sandwiches, wraps, hotdogs, sausages | |  |  |
|  | Hotdogs |  | 2521 |
|  | Luncheon meats |  | 2523 |
|  | Beef bacon, dried beef, pastrami |  | 216 |
|  | Meat and seafood sandwiches and burgers | | 275 |
|  | Meat substitute sandwich |  | 419 |
|  | Nut butter sandwich |  | 423 |
|  | Tomato sandwich |  | 747 |
|  | Sandwich or wrap |  | 58200100, 58200300 |
| Sausage | |  | 2522 and all food codes containing the word "sausage" or "wurst" |
| Soup | |  |  |
|  | Meat and fish soups |  | 283 |
|  | Bean soups |  | 416 |
|  | Potato soups |  | 718 |
|  | Dark-green vegetable soups |  | 723 |
|  | Deep-yellow vegetable soups |  | 735 |
|  | Tomato soups |  | 746 |
|  | Vegetable soups |  | 756 |
|  | Puerto Rican stews and soups |  | 775 |
|  | Soup, other |  | 58400000-58450300 |
| Bread and rolls | |  |  |
|  | Yeast breads and rolls |  | 51 |
|  | Quick breads |  | 52 |
| Sweet baked goods | |  |  |
|  | Coffee cake |  | 536 |
|  | Danishes, breakfast pastries, doughnuts | | 535 |
| Pancakes and waffles | |  |  |
|  | Pancakes |  | 551 |
|  | Waffles |  | 552 |
|  | French toast |  | 553 |
|  | Crepes |  | 554 |
| Oatmeal | |  |  |
|  | Oatmeal |  | 56202900-56203510 |
|  | Cream of wheat |  | 56207000-56207096 |
| Breakfast cereal | |  | 571-574 |
| Pasta | |  |  |
|  | Pasta |  | 561 |
|  | Pasta dishes |  | 58132340-58145800 |
|  | Lasagna, ravioli, spaghetti |  | 58130016-58131600, 58301020, 58302000, 58302060, 58302080, 58304060, 58304200, 58304400 |
| Mexican dishes | |  |  |
|  | Burrito, quesadilla, taco, chimichanga, nachos |  | 58100000-58105100, 58306020, 58306100 |
| Pizza and calzone | |  | 58106200-58108000 |
| Egg dish category | |  | FNDDS code^1^ |
| Whole eggs | |  |  |
|  | Fried, poached, boiled, baked, pickled, deviled |  | 311 and 32102000 |
| Scrambled eggs and omelets | |  | hand code 321 and 324 |
| Egg sandwich | |  |  |
|  | Egg sandwich |  | 322 |
|  | Eggs benedict |  | 32101500 |
| Egg soup | |  | 323 |
| Frozen egg dish | |  | 350 |
| Quiche | |  | 58125120 and 58125180 |
| ^1^Food and Nutrient Database for Dietary Studies, leading numbers in each 8   digit food code | | | |
