# Supplementary material for: Nutrient intake disparities in the US: modeling the effect of food substitutions
Source: Nutr J. 2018 May 17;17:53. doi: 10.1186/s12937-018-0360-z (PMC5960152; doi:10.1186/s12937-018-0360-z)
Supplement: Supplementary file 2 — Table S2. Daily nutrient intake recommendations. (DOCX 26 kb) [file 12937_2018_360_MOESM2_ESM.docx]

| Supplemental Table 2: Daily nutrient intake recommendations | | | | | | | | |  |  |  |  |  |  |  |  |  |  |  |  |  |  |  |  |  |  |
| --- | --- | --- | --- | --- | --- | --- | --- | --- | --- | --- | --- | --- | --- | --- | --- | --- | --- | --- | --- | --- | --- | --- | --- | --- | --- | --- |
|  |  | Male | | | | | | |  | Female, non-pregnant, non-lactating | | | | | | |  | Female, pregnant | | |  | Female, lactating | | |  |  |
| Nutrient |  | 20-30 y |  | 31-50 y |  | 51-70 y |  | 71+ y |  | 20-30 y |  | 31-50 y |  | 51-70 y |  | 71+ y |  | 20-30 y |  | 31-50 y |  | 20-30 y |  | 31-50 y |  |  |
| Saturated fatty acids^1^ (% energy) |  | <10 |  | <10 |  | <10 |  | <10 |  | <10 |  | <10 |  | <10 |  | <10 |  | <10 |  | <10 |  | <10 |  | <10 |  |  |
| Vitamin A (µg)^2,3^ |  | 625 |  | 625 |  | 625 |  | 625 |  | 500 |  | 500 |  | 500 |  | 500 |  | 550 |  | 550 |  | 900 |  | 900 |  |  |
| Vitamin C (mg)^2^ |  | 75 |  | 75 |  | 75 |  | 75 |  | 60 |  | 60 |  | 60 |  | 60 |  | 70 |  | 70 |  | 100 |  | 100 |  |  |
| Vitamin D (µg)^2^ |  | 10 |  | 10 |  | 10 |  | 10 |  | 10 |  | 10 |  | 10 |  | 10 |  | 10 |  | 10 |  | 10 |  | 10 |  |  |
| Vitamin E (mg)^2^ |  | 12 |  | 12 |  | 12 |  | 12 |  | 12 |  | 12 |  | 12 |  | 12 |  | 12 |  | 12 |  | 16 |  | 16 |  |  |
| Thiamin (mg)^2^ |  | 1.0 |  | 1.0 |  | 1.0 |  | 1.0 |  | 0.9 |  | 0.9 |  | 0.9 |  | 0.9 |  | 1.2 |  | 1.2 |  | 1.2 |  | 1.2 |  |  |
| Riboflavin (mg)^2^ |  | 1.1 |  | 1.1 |  | 1.1 |  | 1.1 |  | 0.9 |  | 0.9 |  | 0.9 |  | 0.9 |  | 1.2 |  | 1.2 |  | 1.3 |  | 1.3 |  |  |
| Niacin (mg)^2^ |  | 12 |  | 12 |  | 12 |  | 12 |  | 11 |  | 11 |  | 11 |  | 11 |  | 14 |  | 14 |  | 13 |  | 13 |  |  |
| Vitamin B_6_ (mg)^2^ |  | 1.1 |  | 1.1 |  | 1.4 |  | 1.4 |  | 1.1 |  | 1.1 |  | 1.3 |  | 1.3 |  | 1.6 |  | 1.6 |  | 1.7 |  | 1.7 |  |  |
| Folate (µg)^2,4^ |  | 320 |  | 320 |  | 320 |  | 320 |  | 320 |  | 320 |  | 320 |  | 320 |  | 520 |  | 520 |  | 450 |  | 450 |  |  |
| Vitamin B_12_ (µg)^2^ |  | 2 |  | 2 |  | 2 |  | 2 |  | 2 |  | 2 |  | 2 |  | 2 |  | 2.2 |  | 2.2 |  | 2.4 |  | 2.4 |  |  |
| Calcium (mg)^2^ |  | 800 |  | 800 |  | 800 |  | 1000 |  | 800 |  | 800 |  | 1000 |  | 1000 |  | 800 |  | 800 |  | 800 |  | 800 |  |  |
| Iron (mg)^2^ |  | 6 |  | 6 |  | 6 |  | 6 |  | 8.1 |  | 8.1 |  | 5 |  | 5 |  | 22 |  | 22 |  | 6.5 |  | 6.5 |  |  |
| Magnesium (mg)^2^ |  | 330 |  | 350 |  | 350 |  | 350 |  | 255 |  | 265 |  | 265 |  | 265 |  | 290 |  | 300 |  | 255 |  | 265 |  |  |
| Sodium (mg)^5^ |  | 2300 |  | 2300 |  | 2300 |  | 2300 |  | 2300 |  | 2300 |  | 2300 |  | 2300 |  | 2300 |  | 2300 |  | 2300 |  | 2300 |  |  |
| Zinc (mg)^2^ |  | 9.4 |  | 9.4 |  | 9.4 |  | 9.4 |  | 6.8 |  | 6.8 |  | 6.8 |  | 6.8 |  | 9.5 |  | 9.5 |  | 10.4 |  | 10.4 |  |  |
| Fiber (mg)^6^ |  | 38 |  | 38 |  | 30 |  | 30 |  | 25 |  | 25 |  | 21 |  | 21 |  | 28 |  | 28 |  | 29 |  | 29 |  |  |
| α-linolenic acid (g)^6^ |  | 1.6 |  | 1.6 |  | 1.6 |  | 1.6 |  | 1.1 |  | 1.1 |  | 1.1 |  | 1.1 |  | 1.4 |  | 1.4 |  | 1.3 |  | 1.3 |  |  |
| Vitamin K (µg)^6^ |  | 120 |  | 120 |  | 120 |  | 120 |  | 90 |  | 90 |  | 90 |  | 90 |  | 90 |  | 90 |  | 90 |  | 90 |  |  |
| Choline (mg)^2^ |  | 550 |  | 550 |  | 550 |  | 550 |  | 425 |  | 425 |  | 425 |  | 425 |  | 450 |  | 450 |  | 550 |  | 550 |  |  |
| Potassium (mg)^6^ |  | 4700 |  | 4700 |  | 4700 |  | 4700 |  | 4700 |  | 4700 |  | 4700 |  | 4700 |  | 4700 |  | 4700 |  | 5100 |  | 5100 |  |  |
|  |  |  |  |  |  |  |  |  |  |  |  |  |  |  |  |  |  |  |  |  |  |  |  |  |  |  |
| ^1^US Department of Health and Human Services & US Department of Agriculture. 2015-2020. Dietary Guidelines for Americans 2015-2020. U.S. Government Printing Office, Washington, DC. Available at: http://health.gov/dietaryguidelines/ (verified 29 March 2017) | | | | | | | | | | | | | | | | | | | | | | | | |  |  |
| ^2^Estimated average requirement (EAR). National Academy of Sciences, Institute of Medicine. 2006. Dietary reference intakes: The essential guide to nutrient requirements. Jennifer J. Otten, Jennifer Pitzi Hellwig, Linda D. Meyers (eds.). National Academies Press, Washington, DC. | | | | | | | | | | | | | | | | | | | | | | | | |  |  |
| ^3^Retinol Activity Equivalent (RAE) | | |  |  |  |  |  |  |  |  |  |  |  |  |  |  |  |  |  |  |  |  |  |  |  |  |
| ^4^Dietary Folate Equivalent (DFE) | | |  |  |  |  |  |  |  |  |  |  |  |  |  |  |  |  |  |  |  |  |  |  |  |  |
| ^5^Tolerable upper intake level limit. National Academy of Sciences, Institute of Medicine. 2006. Dietary reference intakes: The essential guide to nutrient requirements. Jennifer J.Otten,   Jennifer Pitzi Hellwig, Linda D. Meyers (eds.). National Academies Press, Washington, DC. | | | | | | | | | | | | | | | | | | | | | | | | |  |  |
| ^6^Adequate Intake (AI). National Academy of Sciences, Institute of Medicine. 2006. Dietary reference intakes: The essential guide to nutrient requirements. Jennifer J. Otten, Jennifer Pitzi Hellwig, Linda D. Meyers (eds.). National Academies Press, Washington, DC. | | | | | | | | | | | | | | | | | | | | | | | | |  |  |
