# Supplementary material for: Nutrient intake disparities in the US: modeling the effect of food substitutions
Source: Nutr J. 2018 May 17;17:53. doi: 10.1186/s12937-018-0360-z (PMC5960152; doi:10.1186/s12937-018-0360-z)
Supplement: Supplementary file 3 — Table S3. Percent of individuals consuming types of main dishes at each eating occasion, 2001–2014 (n = 34,741). (DOCX 23 kb) [file 12937_2018_360_MOESM3_ESM.docx]

| Supplemental Table 3: Percent of individuals consuming types of main dishes at each eating occasion, 2001-2014 (n=34,741) | | | | | | | | | | | | | | |  |  |  |  |
| --- | --- | --- | --- | --- | --- | --- | --- | --- | --- | --- | --- | --- | --- | --- | --- | --- | --- | --- |
|  |  |  |  |  |  |  |  |  |  |  |  |  |  |  |  |  |  |  |
| Food insecure non-participants (n=3,631) | | |  | WIC participants (n=636) | | |  | SNAP participants (n=4,020) | | |  | Food secure non-participants (n=26,454) | | |  |  |  |  |
| \| Breakfast, % (95% CI) \| \| --- \| | | | | | | | | | | | | | | |  |  |  |  |
| Breakfast cereal | 10.9 | (9.4-12.5) |  | Breakfast cereal | 19.8 | (16.3-23.8) |  | Breakfast cereal | 13.3 | (11.9-14.9) |  | Breakfast cereal | 18.8 | (18-19.6) |  |  |  |  |
| Whole eggs | 7.4 | (6.3-8.6) |  | Scrambled eggs | 11.0 | (8.7-13.9) |  | Scrambled eggs | 8.0 | (6.8-9.5) |  | Whole eggs | 7.3 | (6.9-7.8) |  |  |  |  |
| Scrambled eggs | 6.9 | (5.9-8.0) |  | Whole eggs | 5.4 | (3.3-8.5) |  | Whole eggs | 6.4 | (5.4-7.5) |  | Scrambled eggs | 6.8 | (6.3-7.3) |  |  |  |  |
| Sausage | 5.0 | (4.0-6.1) |  | Poultry dish | 4.0 | (2.1-7.3) |  | Sausage | 5.6 | (4.8-6.4) |  | Oatmeal | 5.9 | (5.4-6.3) |  |  |  |  |
| Baked goods | 3.8 | (2.9-4.9) |  | Pancakes and waffles | 3.8 | (2.4-5.8) |  | Bacon | 4.3 | (3.7-5.1) |  | Sausage | 4.5 | (4.1-4.8) |  |  |  |  |
| \| Lunch, % (95% CI) \| \| --- \| | | | | | | | | | | | | | | |  |  |  |  |
| Sandwich | 12.2 | (10.9-13.8) |  | Sandwich | 18.1 | (14.0-23.0) |  | Sandwich | 14.2 | (12.4-16.2) |  | Sandwich | 16.0 | (15.3-16.8) |  |  |  |  |
| Poultry dish | 12.0 | (10.5-13.7) |  | Poultry dish | 12.3 | (8.9-16.8) |  | Poultry dish | 9.1 | (8.0-10.4) |  | Poultry dish | 13.8 | (13.1-14.5) |  |  |  |  |
| Beef dish | 8.9 | (7.7-10.2) |  | Beef dish | 7.6 | (5.3-10.6) |  | Beef dish | 6.9 | (5.9-8.0) |  | Beef dish | 8.5 | (8.0-9.0) |  |  |  |  |
| Soup | 5.1 | (4.1-6.2) |  | Soup | 6.3 | (4.2-9.3) |  | Soup | 4.5 | (3.7-5.4) |  | Soup | 5.7 | (5.3-6.2) |  |  |  |  |
| Seafood dish | 4.8 | (3.9-6.0) |  | Seafood dish | 3.8 | (2-6.9) |  | Seafood dish | 3.4 | (2.8-4.2) |  | Seafood dish | 5.6 | (5.2-6.1) |  |  |  |  |
| \| Dinner, % (95% CI) \| \| --- \| | | | | | | | | | | | | | | |  |  |  |  |
| Poultry dish | 20.5 | (18.5-22.5) |  | Poultry dish | 26.7 | (21.8-32.2) |  | Poultry dish | 21.6 | (19.1-85) |  | Poultry dish | 22.0 | (21.1-23.0) |  |  |  |  |
| Beef dish | 16.2 | (14.4-18.2) |  | Beef dish | 17.5 | (13.6-22.2) |  | Beef dish | 17.6 | (15.8-34.3) |  | Beef dish | 17.9 | (17.1-18.7) |  |  |  |  |
| Sandwich | 9.9 | (8.5-11.4) |  | Sandwich | 12.3 | (8.6-17.3) |  | Sandwich | 9.4 | (8.1-24.3) |  | Seafood dish | 9.4 | (8.7-10.1) |  |  |  |  |
| Seafood dish | 6.5 | (5.3-7.9) |  | Pasta dish | 8.5 | (5.2-13.5) |  | Pasta dish | 7.5 | (5.9-19.6) |  | Sandwich | 9.1 | (8.5-9.7) |  |  |  |  |
| Pork dish | 6.4 | (5.3-7.6) |  | Pizza | 5.9 | (3.5-9.7) |  | Pork dish | 7.1 | (5.8-10.8) |  | Pasta dish | 6.6 | (6.1-7.2) |  |  |  |  |
|  |  |  |  |  |  |  |  |  |  |  |  |  |  |  |  |  |  |  |
| Breakfast cereal: includes all ready-to-eat cereal | | | | |  |  |  |  |  |  |  |  |  |  |  |  |  |  |
| Scrambled eggs: includes all scrambled eggs and omelets | | | | |  |  |  |  |  |  |  |  |  |  |  |  |  |  |
| Whole eggs: includes fried, poached, boiled, baked, pickled, and deviled eggs | | | | | | |  |  |  |  |  |  |  |  |  |  |  |  |
| Poultry dish: includes mixed dishes with poultry and vegetables, frozen or shelf-stable poultry dishes, turkey (except turkey bacon), and duck | | | | | | | | | | | | |  |  |  |  |  |  |
| Pancakes and waffles: includes French toast and crepes | | | | |  |  |  |  |  |  |  |  |  |  |  |  |  |  |
| Sausage: includes all types of sausage | | |  |  |  |  |  |  |  |  |  |  |  |  |  |  |  |  |
| Baked goods: includes coffee cake, danishes, breakfast pastires, and doughnuts | | | | | | |  |  |  |  |  |  |  |  |  |  |  |  |
| Sandwich: includes hotdogs, sasuages, luncheon meats, burgers, wraps, nut butter sandwiches, and tomato sandwiches | | | | | | | | | | |  |  |  |  |  |  |  |  |
| Beef dish: includes mixed dishes with beef and vegetables, frozen or shelf-stable beef dishes, steak, short ribs, roasts, stew meat, brisket, and ground beef | | | | | | | | | | | | | |  |  |  |  |  |
| Soup: includes meat, vegetable, and grain soups; broths; and stews | | | | | |  |  |  |  |  |  |  |  |  |  |  |  |  |
| Seafood dish: includes mixed dishes with seafood and vegetables, seafood salads, and frozen seafood meals | | | | | | | | | |  |  |  |  |  |  |  |  |  |
| Pizza: includes calzones | |  |  |  |  |  |  |  |  |  |  |  |  |  |  |  |  |  |
| Pasta dish: includes lasagna, ravioli, and spaghetti dishes | | | | |  |  |  |  |  |  |  |  |  |  |  |  |  |  |
| Pork dish: includes mixed dishes with pork and vegetables, chops, steaks, ham, roasts, and bacon | | | | | | | | |  |  |  |  |  |  |  |  |  |  |
