# Supplementary material for: Nutrient intake disparities in the US: modeling the effect of food substitutions
Source: Nutr J. 2018 May 17;17:53. doi: 10.1186/s12937-018-0360-z (PMC5960152; doi:10.1186/s12937-018-0360-z)
Supplement: Supplementary file 4 — Table S4. Percent of individuals consuming types of egg dishes at each eating occasion, 2001–2014 (n = 34,741). (DOCX 17 kb) [file 12937_2018_360_MOESM4_ESM.docx]

| Supplemental Table 4: Percent of individuals consuming types of egg dishes at each eating occasion, 2001-2014 (n=34,741) | | | | | | | | | | | | | | |
| --- | --- | --- | --- | --- | --- | --- | --- | --- | --- | --- | --- | --- | --- | --- |
|  |  |  |  |  |  |  |  |  |  |  |  |  |  |  |
| Food insecure non-participants (n=3,631) | | |  | WIC participants (n=636) | | |  | SNAP participants (n=4,020) | | |  | Food secure non-participants (n=26,454) | | |
| \| Breakfast, % (95% CI) \| \| --- \| | | | | | | | | | | | | | | |
| Whole eggs | 7.4 | (6.3-8.6) |  | Scrambled eggs | 11.0 | (8.7-13.9) |  | Scrambled eggs | 8.0 | (6.8-9.5) |  | Whole eggs | 7.3 | (6.9-7.8) |
| Scrambled eggs | 6.9 | (5.9-8.0) |  | Whole eggs | 5.4 | (3.3-8.5) |  | Whole eggs | 6.4 | (5.4-7.5) |  | Scrambled eggs | 6.8 | (6.3-7.3) |
| Egg sandwich | 1.2 | (0.8-1.7) |  | Egg sandwich | 1.6 | (0.6-4.1) |  | Egg sandwich | 0.8 | (0.5-1.2) |  | Egg sandwich | 1.1 | (0.9-1.4) |
| \| Lunch, % (95% CI) \| \| --- \| | | | | | | | | | | | | | | |
| Whole eggs | 1.2 | (0.8-1.8) |  | Whole eggs | 3.1 | (1.5-6.6) |  | Scrambled eggs | 1.8 | (1.6-2.0) |  | Whole eggs | 1.6 | (1.4-1.8) |
| Scrambled eggs | 1.0 | (0.8-1.4) |  | Scrambled eggs | 2.5 | (1.3-4.8) |  | Whole eggs | 0.9 | (0.8-1.0) |  | Scrambled eggs | 1.2 | (1.0-1.4) |
| Egg soup | <0.1 | (0.0-0.2) |  | Egg sandwich | 0.6 | (0.2-2.4) |  | Egg sandwich | 0.3 | (0.2-0.3) |  | Egg soup | 0.1 | (0.1-0.2) |
| \| Dinner, % (95% CI) \| \| --- \| | | | | | | | | | | | | | | |
| Whole eggs | 1.6 | (1.1-2.4) |  | Scrambled eggs | 1.6 | (0.8-3.0) |  | Whole eggs | 1.8 | (1.4-1.8) |  | Whole eggs | 1.7 | (1.5-2.0) |
| Scrambled eggs | 1.3 | (0.9-1.8) |  | Whole eggs | 0.8 | (0.4-1.5) |  | Scrambled eggs | 1.1 | (1.0-2.0) |  | Scrambled eggs | 1.1 | (0.9-1.3) |
| Egg soup | 0.2 | (0.0-1.2) |  | Egg soup | 0.3 | (0.0-0.2) |  | Egg soup | <0.1 | (0.0-0.1) |  | Egg soup | 0.2 | (0.1-0.2) |
|  |  |  |  |  |  |  |  |  |  |  |  |  |  |  |
| Scrambled eggs: includes all scrambled eggs and omelets | | | | |  |  |  |  |  |  |  |  |  |  |
| Whole eggs: includes fried, poached, boiled, baked, pickled, and deviled eggs | | | | | | |  |  |  |  |  |  |  |  |
| Egg sandwich: includes eggs benedict | | | |  |  |  |  |  |  |  |  |  |  |  |
| Egg soup: represents egg drop soup | | |  |  |  |  |  |  |  |  |  |  |  |  |
