# Supplementary material for: Nutrient intake disparities in the US: modeling the effect of food substitutions
Source: Nutr J. 2018 May 17;17:53. doi: 10.1186/s12937-018-0360-z (PMC5960152; doi:10.1186/s12937-018-0360-z)
Supplement: Supplementary file 5 — Table S5. Amount of each dish used in substitution modeling. (DOCX 21 kb) [file 12937_2018_360_MOESM5_ESM.docx]

| Supplemental Table 5: Amount of each dish used in substitution modeling | | | | | | | | | | | | |
| --- | --- | --- | --- | --- | --- | --- | --- | --- | --- | --- | --- | --- |
|  |  |  |  |  |  |  |  |  |  |  |  |  |
| Dish |  | Food insecure non-participants (n=3,631) | |  | WIC participants (n=636) | |  | SNAP participants (n=4,020) | |  | Food secure non-participants (n=26,454) | |
|  |  | \| Mean (95% CI), grams \| \| --- \| | | | | | | | | | | |
| Breakfast cereal |  | 40 | (30-49) |  | 60 | (34-86) |  | 71 | (47-94)* |  | 50 | (43-57) |
| Sandwich |  | 162 | (148-176) |  | 142 | (116-168) |  | 139 | (130-148)* |  | 137 | (133-142)** |
| Poultry dish |  | 110 | (80-140) |  | 151 | (106-196) |  | 132 | (115-149) |  | 107 | (98-116) |
|  |  |  |  |  |  |  |  |  |  |  |  |  |
| Breakfast cereal: includes all ready-to-eat cereal | | | |  |  |  |  |  |  |  |  |  |
| Sandwich: includes hotdogs, sasuages, luncheon meats, burgers, wraps, nut butter sandwiches, and tomato sandwiches | | | | | | | | | | | | |
| Poultry dish: includes mixed dishes with poultry and vegetables, frozen or shelf-stable poultry dishes, turkey (except turkey bacon), and duck | | | | | | | | | | | | |
| Scrambled eggs: includes all scrambled eggs and omelets | | | | | |  |  |  |  |  |  |  |
| Whole eggs: includes fried, poached, boiled, baked, pickled, and deviled eggs | | | | | | |  |  |  |  |  |  |
| *Different than food insecure non-participants at P<0.016 (Bonferroni adjusted for multiple comparisons) | | | | | | | | | | | |  |
| **Different than food insecure non-participants at P<0.003 (Bonferroni adjusted for multiple comparisons) | | | | | | | | | | | |  |
|  |  |  |  |  |  |  |  |  |  |  |  |  |
