# Supplementary material for: Nutrient intake disparities in the US: modeling the effect of food substitutions
Source: Nutr J. 2018 May 17;17:53. doi: 10.1186/s12937-018-0360-z (PMC5960152; doi:10.1186/s12937-018-0360-z)
Supplement: Supplementary file 6 — Table S6. Nutrient content of dishes used in substitution modeling. (DOCX 32 kb) [file 12937_2018_360_MOESM6_ESM.docx]

| Supplemental Table 6: Nutrient content of dishes used in substitution modeling | | | | | | |  |  |  |  |  |  |  |  |  |  |  |  |  |  |  |  |  |  |  |
| --- | --- | --- | --- | --- | --- | --- | --- | --- | --- | --- | --- | --- | --- | --- | --- | --- | --- | --- | --- | --- | --- | --- | --- | --- | --- |
| Nutrient |  | Breakfast cereal | |  | Sandwiches | |  | Poultry dishes | |  | Scrambled eggs | |  | Whole eggs | |  |  |  |  |  |  |  |  |  |  |
|  |  | Mean, per gram of dish (95% CI) | | | | | | | | | | | | | |  |  |  |  |  |  |  |  |  |  |
| Energy (kcal) |  | 3.688 | (3.678-3.698) |  | 2.015 | (1.991-2.039) |  | 2.047 | (2.033-2.062) |  | 1.720 | (1.705-1.735) |  | 1.757 | (1.741-1.773) |  |  |  |  |  |  |  |  |  |  |
| Protein, total (g) |  | 0.088 | (0.087-0.089) |  | 0.156 | (0.155-0.157) |  | 0.223 | (0.221-0.225) |  | 0.116 | (0.116-0.117) |  | 0.131 | (0.130-0.131) |  |  |  |  |  |  |  |  |  |  |
| Carhobydrate, total (g) |  | 0.803 | (0.801-0.805) |  | 0.096 | (0.093-0.098) |  | 0.054 | (0.053-0.056) |  | 0.022 | (0.022-0.023) |  | 0.009 | (0.009-0.009) |  |  |  |  |  |  |  |  |  |  |
| Fiber (mg) |  | 0.077 | (0.075-0.078) |  | 0.005 | (0.005-0.005) |  | 0.004 | (0.004-0.004) |  | 0.001 | (0.001-0.001) |  | <0.001 |  |  |  |  |  |  |  |  |  |  |  |
| Fatty acids, total (g) |  | 0.040 | (0.039-0.041) |  | 0.107 | (0.104-0.109) |  | 0.099 | (0.097-0.100) |  | 0.128 | (0.126-0.130) |  | 0.129 | (0.128-0.131) |  |  |  |  |  |  |  |  |  |  |
| Saturated fatty acids (g) |  | 0.008 | (0.008-0.008) |  | 0.037 | (0.036-0.038) |  | 0.024 | (0.024-0.025) |  | 0.042 | (0.041-0.042) |  | 0.039 | (0.038-0.039) |  |  |  |  |  |  |  |  |  |  |
| Eicosapentaenoic acid (g) |  | <0.001 |  |  | <0.001 |  |  | <0.001 |  |  | <0.001 |  |  | <0.001 |  |  |  |  |  |  |  |  |  |  |  |
| Docosahexaenoic acid (g) |  | <0.001 |  |  | <0.001 |  |  | <0.001 |  |  | <0.001 |  |  | <0.001 |  |  |  |  |  |  |  |  |  |  |  |
| Vitamin A (µg)^1^ |  | 4.870 | (4.790-4.950) |  | 0.095 | (0.091-0.099) |  | 0.245 | (0.239-0.251) |  | 1.396 | (1.380-1.412) |  | 1.667 | (1.649-1.685) |  |  |  |  |  |  |  |  |  |  |
| Vitamin C (mg) |  | 0.215 | (0.208-0.223) |  | 0.010 | (0.009-0.010) |  | 0.010 | (0.010-0.011) |  | 0.009 | (0.008-0.011) |  | <0.001 |  |  |  |  |  |  |  |  |  |  |  |
| Vitamin D (µg) |  | 0.027 | (0.026-0.027) |  | 0.004 | (0.004-0.004) |  | 0.001 | (0.001-0.001) |  | 0.015 | (0.015-0.016) |  | 0.019 | (0.019-0.020) |  |  |  |  |  |  |  |  |  |  |
| Vitamin E (mg) |  | 0.033 | (0.031-0.034) |  | 0.003 | (0.003-0.003) |  | 0.006 | (0.006-0.006) |  | 0.011 | (0.011-0.011) |  | 0.013 | (0.013-0.013) |  |  |  |  |  |  |  |  |  |  |
| Thiamin (mg) |  | 0.014 | (0.014-0.014) |  | 0.002 | (0.002-0.003) |  | 0.001 | (0.001-0.001) |  | 0.001 | (0.001-0.001) |  | 0.001 | (0.001-0.001) |  |  |  |  |  |  |  |  |  |  |
| Riboflavin (mg) |  | 0.015 | (0.015-0.015) |  | 0.002 | (0.002-0.002) |  | 0.002 | (0.002-0.002) |  | 0.004 | (0.004-0.004) |  | 0.005 | (0.005-0.005) |  |  |  |  |  |  |  |  |  |  |
| Niacin (mg) |  | 0.180 | (0.177-0.183) |  | 0.044 | (0.044-0.045) |  | 0.071 | (0.071-0.072) |  | 0.003 | (0.003-0.003) |  | 0.001 | (0.001-0.001) |  |  |  |  |  |  |  |  |  |  |
| Vitamin B_6_ (mg) |  | 0.022 | (0.022-0.022) |  | 0.003 | (0.003-0.003) |  | 0.004 | (0.004-0.004) |  | 0.001 | (0.001-0.001) |  | 0.001 | (0.001-0.001) |  |  |  |  |  |  |  |  |  |  |
| Folate (µg)^2^ |  | 9.119 | (8.968-9.269) |  | 0.273 | (0.265-0.282) |  | 0.151 | (0.148-0.155) |  | 0.272 | (0.269-0.274) |  | 0.382 | (0.378-0.386) |  |  |  |  |  |  |  |  |  |  |
| Vitamin B_12_ (µg) |  | 0.060 | (0.059-0.061) |  | 0.008 | (0.008-0.008) |  | 0.003 | (0.003-0.003) |  | 0.008 | (0.008-0.008) |  | 0.010 | (0.010-0.010) |  |  |  |  |  |  |  |  |  |  |
| Calcium (mg) |  | 1.824 | (1.743-1.906) |  | 0.471 | (0.458-0.484) |  | 0.196 | (0.191-0.201) |  | 0.885 | (0.868-0.903) |  | 0.555 | (0.550-0.560) |  |  |  |  |  |  |  |  |  |  |
| Iron (mg) |  | 0.216 | (0.212-0.219) |  | 0.015 | (0.014-0.015) |  | 0.010 | (0.010-0.010) |  | 0.013 | (0.013-0.014) |  | 0.017 | (0.017-0.017) |  |  |  |  |  |  |  |  |  |  |
| Magnesium (mg) |  | 0.811 | (0.795-0.826) |  | 0.194 | (0.193-0.196) |  | 0.232 | (0.231-0.233) |  | 0.135 | (0.135-0.136) |  | 0.120 | (0.119-0.121) |  |  |  |  |  |  |  |  |  |  |
| Sodium (mg) |  | 5.080 | (5.003-5.158) |  | 9.101 | (8.994-9.209) |  | 4.072 | (4.029-4.116) |  | 3.653 | (3.604-3.702) |  | 3.600 | (3.552-3.649) |  |  |  |  |  |  |  |  |  |  |
| Zinc (mg) |  | 0.084 | (0.082-0.087) |  | 0.019 | (0.019-0.020) |  | 0.014 | (0.013-0.014) |  | 0.012 | (0.011-0.012) |  | 0.012 | (0.012-0.012) |  |  |  |  |  |  |  |  |  |  |
| α-linolenic acid (g) |  | 0.001 | (0.001-0.001) |  | 0.002 | (0.001-0.002) |  | 0.002 | (0.002-0.002) |  | 0.002 | (0.002-0.002) |  | 0.001 | (0.001-0.002) |  |  |  |  |  |  |  |  |  |  |
| Vitamin K (µg) |  | 0.017 | (0.016-0.017) |  | 0.029 | (0.028-0.029) |  | 0.055 | (0.054-0.057) |  | 0.051 | (0.048-0.055) |  | 0.031 | (0.029-0.032) |  |  |  |  |  |  |  |  |  |  |
| Choline (mg) |  | 0.210 | (0.206-0.214) |  | 0.483 | (0.478-0.488) |  | 0.571 | (0.566-0.577) |  | 1.707 | (1.686-1.728) |  | 2.448 | (2.423-2.473) |  |  |  |  |  |  |  |  |  |  |
| Potassium (mg) |  | 3.236 | (3.181-3.291) |  | 2.989 | (2.943-3.036) |  | 2.446 | (2.429-2.462) |  | 1.604 | (1.593-1.615) |  | 1.415 | (1.411-1.419) |  |  |  |  |  |  |  |  |  |  |
| α-carotene (µg) |  | 0.070 | (0.066-0.074) |  | 0.019 | (0.018-0.020) |  | 0.201 | (0.182-0.219) |  | 0.013 | (0.011-0.016) |  | 0.003 | (0.002-0.004) |  |  |  |  |  |  |  |  |  |  |
| β-carotene (µg) |  | 0.255 | (0.218-0.292) |  | 0.202 | (0.193-0.211) |  | 0.655 | (0.607-0.703) |  | 0.313 | (0.280-0.345) |  | 0.139 | (0.132-0.147) |  |  |  |  |  |  |  |  |  |  |
| β-cryptoxanthin (µg) |  | 0.029 | (0.026-0.032) |  | 0.011 | (0.010-0.012) |  | 0.017 | (0.015-0.018) |  | 0.064 | (0.063-0.065) |  | 0.093 | (0.092-0.094) |  |  |  |  |  |  |  |  |  |  |
| Lycopene (µg) |  | 0.024 | (0.003-0.046) |  | 1.568 | (1.467-1.669) |  | 1.877 | (1.686-2.067) |  | 0.254 | (0.203-0.305) |  | <0.001 |  |  |  |  |  |  |  |  |  |  |  |
| Lutein and   zeaxanthin (µg) |  | 2.047 | (1.967-2.126) |  | 0.172 | (0.164-0.179) |  | 0.373 | (0.350-0.395) |  | 3.016 | (2.947-3.085) |  | 4.163 | (4.103-4.222) |  |  |  |  |  |  |  |  |  |  |
|  |  |  |  |  |  |  |  |  |  |  |  |  |  |  |  |  |  |  |  |  |  |  |  |  |  |
| 95% CI not listed if nutrient content is <0.001 units. | | | |  |  |  |  |  |  |  |  |  |  |  |  |  |  |  |  |  |  |  |  |  |  |
| Breakfast cereal: includes all ready-to-eat cereal | | | |  |  |  |  |  |  |  |  |  |  |  |  |  |  |  |  |  |  |  |  |  |  |
| Sandwich: includes hotdogs, sasuages, luncheon meats, burgers, wraps, nut butter sandwiches, and tomato sandwiches | | | | | | | | | |  |  |  |  |  |  |  |  |  |  |  |  |  |  |  |  |
| Poultry dish: includes mixed dishes with poultry and vegetables, frozen or shelf-stable poultry dishes, turkey (except turkey bacon), and duck | | | | | | | | | | | | |  |  |  |  |  |  |  |  |  |  |  |  |  |
| Scrambled eggs: includes all scrambled eggs and omelets | | | |  |  |  |  |  |  |  |  |  |  |  |  |  |  |  |  |  |  |  |  |  |  |
| Whole eggs: includes fried, poached, boiled, baked, pickled, and deviled eggs | | | | | |  |  |  |  |  |  |  |  |  |  |  |  |  |  |  |  |  |  |  |  |
| ^1^Retinol Activity Equivalent (RAE) | | |  |  |  |  |  |  |  |  |  |  |  |  |  |  |  |  |  |  |  |  |  |  |  |
| ^2^Dietary Folate Equivalent (DFE) | |  |  |  |  |  |  |  |  |  |  |  |  |  |  |  |  |  |  |  |  |  |  |  |  |
