# Supplementary material for: Nutrient intake disparities in the US: modeling the effect of food substitutions
Source: Nutr J. 2018 May 17;17:53. doi: 10.1186/s12937-018-0360-z (PMC5960152; doi:10.1186/s12937-018-0360-z)
Supplement: Supplementary file 7 — Table S7. Sensitivity analysis for total nutrient index. (DOCX 27 kb) [file 12937_2018_360_MOESM7_ESM.docx]

| Supplemental Table 7: Sensitivity analysis for total nutrient index | | | | | |  |  |  |  |  |  |  | | | |  | | | | | | | | | | | | | |  | | | |  | | | | | | | | |  | | | | | | | | | | | |  | | | | |  | | | | |  | | | | |  | | | | |  | | | | |  | | | | |  | | | | |  | | | | |  | | | | |  | | | | |  | | | | |  | | |
| --- | --- | --- | --- | --- | --- | --- | --- | --- | --- | --- | --- | --- | --- | --- | --- | --- | --- | --- | --- | --- | --- | --- | --- | --- | --- | --- | --- | --- | --- | --- | --- | --- | --- | --- | --- | --- | --- | --- | --- | --- | --- | --- | --- | --- | --- | --- | --- | --- | --- | --- | --- | --- | --- | --- | --- | --- | --- | --- | --- | --- | --- | --- | --- | --- | --- | --- | --- | --- | --- | --- | --- | --- | --- | --- | --- | --- | --- | --- | --- | --- | --- | --- | --- | --- | --- | --- | --- | --- | --- | --- | --- | --- | --- | --- | --- | --- | --- | --- | --- | --- | --- | --- | --- | --- | --- | --- | --- | --- | --- | --- | --- | --- |
| Nutrient | |  | Model 1 | |  | Model 2 | |  | Model 3 | |  | Model 4 | | | | | | | | | | | | | | | | | |  | | | | Model 5 | | | | | | | | | | | | | | | | | | | | |  | | | | |  | | | | |  | | | | |  | | | | |  | | | | |  | | | | |  | | | | |  | | | | |  | | | | |  | | | | |  | | | | |  | | |
|  |  |  | Total nutrient index (95% CI)^1^ | | | | | | | | | | | | | | | | | | | | | | | | | | | | | | | | | | | | | | | | | | | | | | | | | | | |  | | | | |  | | | | |  | | | | |  | | | | |  | | | | |  | | | | |  | | | | |  | | | | |  | | | | |  | | | | |  | | | | |  | | |
| Food insecure non-participants | |  |  |  |  |  |  |  |  |  |  |  | | | |  | | | | | | | | | | | | | |  | | | |  | | | | | | | | |  | | | | | | | | | | | |  | | | | |  | | | | |  | | | | |  | | | | |  | | | | |  | | | | |  | | | | |  | | | | |  | | | | |  | | | | |  | | | | |  | | |
|  | Breakfast |  | <0.001 | |  | <0.001 | |  | <0.001 | |  | <0.001 | | | | | | | | | | | | | | | | | |  | | | | <0.001 | | | | | | | | | | | | | | | | | | | | |  | | | | |  | | | | |  | | | | |  | | | | |  | | | | |  | | | | |  | | | | |  | | | | |  | | | | |  | | | | |  | | | | |  | | |
|  | Lunch |  | -0.39 | (-1.95, 1.17) |  | -0.44 | (-1.95, 1.17) |  | -0.39 | (-1.91, 1.13) |  | -0.42 | | | | (-1.98, 1.15) | | | | | | | | | | | | | |  | | | | -0.37 | | | | | | | | | (-2.07, 1.34) | | | | | | | | | | | |  | | | | |  | | | | |  | | | | |  | | | | |  | | | | |  | | | | |  | | | | |  | | | | |  | | | | |  | | | | |  | | | | |  | | |
|  | Dinner |  | -0.25 | (-2.48, 1.99) |  | -0.38 | (-2.48, 1.99) |  | -0.34 | (-2.50, 1.83) |  | -0.32 | | | | (-2.40, 1.76) | | | | | | | | | | | | | |  | | | | -0.14 | | | | | | | | | (-2.52, 2.24) | | | | | | | | | | | |  | | | | |  | | | | |  | | | | |  | | | | |  | | | | |  | | | | |  | | | | |  | | | | |  | | | | |  | | | | |  | | | | |  | | |
| SNAP participants | |  |  |  |  |  |  |  |  |  |  |  | | | |  | | | | | | | | | | | | | |  | | | |  | | | | | | | | |  | | | | | | | | | | | |  | | | | |  | | | | |  | | | | |  | | | | |  | | | | |  | | | | |  | | | | |  | | | | |  | | | | |  | | | | |  | | | | |  | | |
|  | Breakfast |  | -0.63 | (-1.74, 0.49) |  | -0.72 | (-1.84, 0.40) |  | -0.55 | (-1.58, 0.48) |  | -0.62 | | | | (-1.77, 0.52) | | | | | | | | | | | | | |  | | | | -0.60 | | | | | | | | | (-1.76, 0.56) | | | | | | | | | | | |  | | | | |  | | | | |  | | | | |  | | | | |  | | | | |  | | | | |  | | | | |  | | | | |  | | | | |  | | | | |  | | | | |  | | |
|  | Lunch |  | 0.01 | (-1.43, 1.45) |  | -0.06 | (-1.49, 1.37) |  | 0.00 | (-1.40, 1.39) |  | -0.03 | | | | (-1.46, 1.39) | | | | | | | | | | | | | |  | | | | 0.07 | | | | | | | | | (-1.49, 1.63) | | | | | | | | | | | |  | | | | |  | | | | |  | | | | |  | | | | |  | | | | |  | | | | |  | | | | |  | | | | |  | | | | |  | | | | |  | | | | |  | | |
|  | Dinner |  | -0.08 | (-1.98, 1.83) |  | -0.19 | (-2.04, 1.66) |  | -0.18 | (-1.99, 1.63) |  | -0.12 | | | | (-1.93, 1.69) | | | | | | | | | | | | | |  | | | | 0.05 | | | | | | | | | (-1.98, 2.09) | | | | | | | | | | | |  | | | | |  | | | | |  | | | | |  | | | | |  | | | | |  | | | | |  | | | | |  | | | | |  | | | | |  | | | | |  | | | | |  | | |
| WIC participants | |  |  |  |  |  |  |  |  |  |  |  | | | |  | | | | | | | | | | | | | |  | | | |  | | | | | | | | |  | | | | | | | | | | | |  | | | | |  | | | | |  | | | | |  | | | | |  | | | | |  | | | | |  | | | | |  | | | | |  | | | | |  | | | | |  | | | | |  | | |
|  | Breakfast |  | -1.14 | (-2.75, 0.47) |  | -1.27 | (-2.87, 0.34) |  | -1.04 | (-2.54, 0.45) |  | -1.12 | | | | (-2.78, 0.54) | | | | | | | | | | | | | |  | | | | -1.12 | | | | | | | | | (-2.81, 0.57) | | | | | | | | | | | |  | | | | |  | | | | |  | | | | |  | | | | |  | | | | |  | | | | |  | | | | |  | | | | |  | | | | |  | | | | |  | | | | |  | | |
|  | Lunch |  | -0.63 | (-3.54, 2.28) |  | -0.73 | (-3.64, 2.17) |  | -0.61 | (-3.44, 2.21) |  | -0.68 | | | | (-3.36, 2.30) | | | | | | | | | | | | | |  | | | | -0.58 | | | | | | | | | (-3.76, 2.60) | | | | | | | | | | | |  | | | | |  | | | | |  | | | | |  | | | | |  | | | | |  | | | | |  | | | | |  | | | | |  | | | | |  | | | | |  | | | | |  | | |
|  | Dinner |  | 0.00 | (-1.92, 1.92) |  | -0.10 | (-1.99, 1.79) |  | -0.12 | (-2.00, 1.76) |  | -0.03 | | | | (-1.88, 1.82) | | | | | | | | | | | | | |  | | | | 0.14 | | | | | | | | | (-1.91, 2.19) | | | | | | | | | | | |  | | | | |  | | | | |  | | | | |  | | | | |  | | | | |  | | | | |  | | | | |  | | | | |  | | | | |  | | | | |  | | | | |  | | |
| Food secure non-participants | |  |  |  |  |  |  |  |  |  |  |  | | | |  | | | | | | | | | | | | | |  | | | |  | | | | | | | | |  | | | | | | | | | | | |  | | | | |  | | | | |  | | | | |  | | | | |  | | | | |  | | | | |  | | | | |  | | | | |  | | | | |  | | | | |  | | | | |  | | |
|  | Breakfast |  | -0.42 | (-1.41, 0.57) |  | -0.46 | (-1.45, 0.54) |  | -0.40 | (-1.35, 0.55) |  | -0.43 | | | | (-1.44, 0.58) | | | | | | | | | | | | | |  | | | | -0.40 | | | | | | | | | (-1.41, 0.62) | | | | | | | | | | | |  | | | | |  | | | | |  | | | | |  | | | | |  | | | | |  | | | | |  | | | | |  | | | | |  | | | | |  | | | | |  | | | | |  | | |
|  | Lunch |  | -0.34 | (-2.44, 1.76) |  | -0.41 | (-2.51, 1.70) |  | -0.36 | (-2.40, 1.69) |  | -0.39 | | | | (-2.49, 1.71) | | | | | | | | | | | | | |  | | | | -0.30 | | | | | | | | | (-2.59, 1.99) | | | | | | | | | | | |  | | | | |  | | | | |  | | | | |  | | | | |  | | | | |  | | | | |  | | | | |  | | | | |  | | | | |  | | | | |  | | | | |  | | |
|  | Dinner |  | -0.23 | (-2.00, 1.54) |  | -0.34 | (-2.05, 1.36) |  | -0.32 | (-2.02, 1.37) |  | -0.27 | | | | (-1.98, 1.44) | | | | | | | | | | | | | |  | | | | -0.14 | | | | | | | | | (-2.02, 1.75) | | | | | | | | | | | |  | | | | |  | | | | |  | | | | |  | | | | |  | | | | |  | | | | |  | | | | |  | | | | |  | | | | |  | | | | |  | | | | |  | | |
|  |  |  |  |  |  |  |  |  |  |  |  |  | | | |  | | | | | | | | | | | | | |  | | | |  | | | | | | | | |  | | | | | | | | | | | |  | | | | |  | | | | |  | | | | |  | | | | |  | | | | |  | | | | |  | | | | |  | | | | |  | | | | |  | | | | |  | | | | |  | | |
| ^1^Represents mean percent change in nutrient intake from baseline, averaged across all nutrients, weighted by the inverse of the standard error of each nutrient. 95% CI not listed if value <0.001. | | | | | | | | | | | | | | | | | | | | | | | | | | | | | | | | | |  | | | | | | | | | | | | | | |  | | | | | | | |  | | | |  | | | | |  | | | | |  | | | | |  | | | | |  | | | | |  | | | | |  | | | | |  | | | | |  | | | | |  |  |  |  |  |  |  |
| Model 1: total protein, total carbohydrates, fiber, total fat, saturated fat, vitamin A, vitamin C, vitamin D, vitamin E, vitamin K, thiamin, riboflavin, niacin, vitamin B6, folate, vitamin B12, calcium, iron, magnesium, sodium, zinc, eicosapentanoic acid, docosahexanoic acid, α-linoleic acid, choline, potassium, α-carotene, β-carotene, lutein and zeaxanthin, and lycopene. | | | | | | | | | | | | | | | | | | | | | | | | | | | | | | | | | |  | | | | | | | | | | | | | | |  | | | | | | | |  | | | |  | | | | |  | | | | |  | | | | |  | | | | |  | | | | |  | | | | |  | | | | |  | | | | |  | | | | |  |  |  |  |  |  |  |
| Model 2: Model 1 without choline. | | | | | | | | | | | | |  |  |  | |  | |  | |  | |  | |  | |  | | | |  | | | |  | | |  | | |  | | | |  | | |  | | |  | | | | | | | |  | | | | |  | | | | |  | | | | |  | | | |  | | | | |  | | | | |  | | | | |  | | | | |  | | | | |  | | | | |  | | | | |
| Model 3: Model 1 without folate. | | | | | | | | | | | | |  |  |  | |  | |  | |  | |  | |  | |  | | | |  | | | |  | | |  | | |  | | | |  | | |  | | |  | | | | | | | |  | | | | |  | | | | |  | | | | |  | | | |  | | | | |  | | | | |  | | | | |  | | | | |  | | | | |  | | | | |  | | | | |
| Model 4: Model 1 without vitamin D. | | | | | | | | | | | | |  |  |  | |  | |  | |  | |  | |  | |  | | | |  | | | |  | | |  | | |  | | | |  | | |  | | |  | | | | | | | |  | | | | |  | | | | |  | | | | |  | | | |  | | | | |  | | | | |  | | | | |  | | | | |  | | | | |  | | | | |  | | | | |
| Model 5: Model 1 without magnesium. | | | | | | | | | | | | | |  |  | |  | |  | |  | |  | |  | |  | | | |  | | | |  | | |  | | |  | | | |  | | |  | | |  | | | | | | | |  | | | | |  | | | | |  | | | | |  | | | |  | | | | |  | | | | |  | | | | |  | | | | |  | | | | |  | | | | |  | | | | |
| SNAP, Supplemental Nutrition Assistance Program | | | | | | | | | | | | | | | |  | |  | |  | |  | |  | |  | |  | | | |  | | | |  | | |  | | |  | | | |  | | | |  | | | | | | | |  | | | | |  | | | |  | | | | |  | | | | |  | | | | |  | | | | |  | | | | |  | | | | |  | | | | |  | | | | |  | | | | |  |
| WIC, Special Supplemental Nutrition Program for Women, Infants, and Children | | | | | | | | | | | | | | | | | | | | | | | | | | | | |  | | | |  | | | |  | | |  | | | |  | | |  | | | | |  |  |  | |  | | | | | |  | | | | | |  | | | | |  | | | | | |  | | | | |  | | | | |  | | | | |  | | | | |  | | | | |  | | | | |  | |  | |
